# Novel Phthalazin-1(2H)-One Derivatives Displaying a Dithiocarbamate Moiety as Potential Anticancer Agents

**DOI:** 10.3390/molecules27238115

**Published:** 2022-11-22

**Authors:** Noemí Vila, Pedro Besada, José Brea, María Isabel Loza, Carmen Terán

**Affiliations:** 1 Departamento de Química Orgánica, Universidade de Vigo, 36310 Vigo, Spain; 2 Instituto de Investigación Sanitaria Galicia Sur, Hospital Álvaro Cunqueiro, 36213 Vigo, Spain; 3 Drug Screening Platform/Biofarma Research Group, CIMUS Research Center, Departamento de Farmacoloxía, Farmacia e Tecnoloxía Farmacéutica, Universidade de Santiago de Compostela, 15782 Santiago de Compostela, Spain

**Keywords:** phthalazinone, dithiocarbamate, hybridization, one-pot synthesis, antiproliferative activity, drug-likeness, toxicity

## Abstract

Nowadays, cancer disease seems to be the second most common cause of death worldwide. Molecular hybridization is a drug design strategy that has provided promising results against multifactorial diseases, including cancer. In this work, two series of phthalazinone-dithiocarbamate hybrids were described, compounds **6**–**8**, which display the dithiocarbamate scaffold at N2, and compounds **9**, in which this moiety was placed at C4. The proposed compounds were successfully synthesized via the corresponding aminoalkyl phthalazinone derivatives and using a one-pot reaction with carbon disulfide, anhydrous H_3_PO_4_, and different benzyl or propargyl bromides. The antiproliferative effects of the titled compounds were explored against three human cancer cell lines (A2780, NCI-H460, and MCF-7). The preliminary results revealed significant differences in activity and selectivity depending on the dithiocarbamate moiety location. Thus, in general terms, compounds **6***–***8** displayed better activity against the A-2780 and MCF-7 cell lines, while most of the analogues of the **9** group were selective toward the NCI-H460 cell line. Compounds **6e**, **8e**, **6g**, **9a**–**b**, **9d**, and **9g** with IC_50_ values less than 10 µM were the most promising. The drug-likeness and toxicity properties of the novel phthalazinone-dithiocarbamate hybrids were predicted using Swiss-ADME and ProTox web servers, respectively.

## 1. Introduction

Nowadays, cancer disease seems to be the major cause of death in high-income and upper-middle-income countries, and the second most common cause of death worldwide [1]. It is estimated that almost 10 million people died from cancer in 2020, and 19.3 million new cases were diagnosed [2]. The toxicity and the side effects of current antineoplastic drugs, as well as the appearance of drug resistance, are the main drawbacks of chemotherapy. Therefore, developing new small molecules with fewer side effects, low toxicity and higher efficiency toward cancer is a challenge for researchers [3,4].

The molecular hybridization strategy is based on the combination of pharmacophores from different bioactive molecules in new hybrid compounds. This interesting approach has received significant attention in rational drug design since it would allow derivatives to be obtained with improved affinity and efficacy and often overcome cross-resistance [5]. In addition, in some cases, this approach provided compounds showing modified selectivity profiles, reduced side effects, or even different or dual action mechanisms. Researchers have reported promising results using the molecular hybridization tool against multifactorial diseases, including cancer [6].

**Figure 1:**
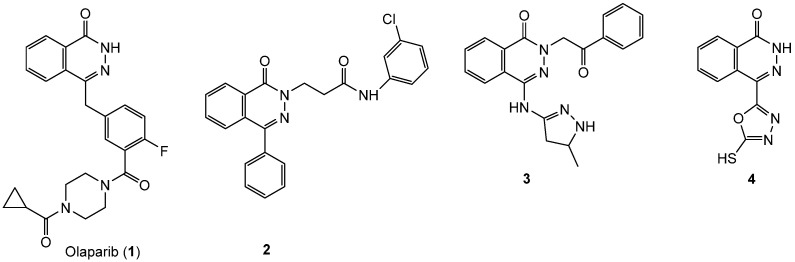
Structure of several phthalazinone derivatives with anticancer properties.

Phthalazin-1(2H)-one core is a privileged building block for drug development, since phthalazinone derivatives possess a wide spectrum of pharmacological activities, including anticancer, antidiabetic, antiasthmatic, antihistaminic, antihypertensive, antithrombotic, antidepressant, anti-inflammatory and analgesic effects [7,8,9]. Several 4-substituted phthalazinones, such as olaparib (**1**) and compound **2** (Figure 1), are recognized antitumor agents acting through poly ADP ribose polymerase (PARP) inhibition [10,11,12]; pyrazole-phthalazinone hybrids, such as compound **3**, are also promising drug candidates for cancer treatment, acting as aurora kinase inhibitors [13]. In addition, the anticancer activities of oxadiazol-phthalazinones, such as compound **4**, seem to be related to their p38 mitogen-activated protein kinase (MAPK) and topoisomerase II inhibition [14].

**Figure 2:**
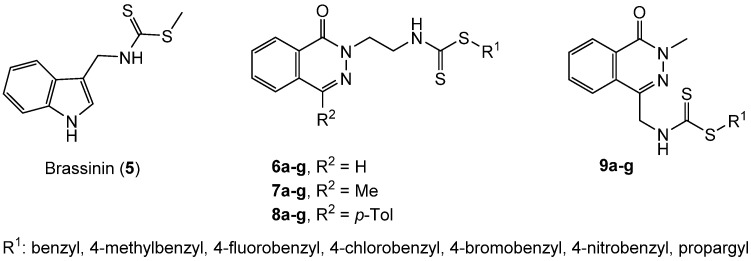
Brassinin (**5**) and the novel phthalazinone-dithiocarbamate hybrids (compounds **6**–**9**).

Dithiocarbamate is another chemical entity with a wide range of biological properties, also including anticancer activity [15,16]. The natural product brassinin (**5**) and related indole-dithiocarbamates are inhibitors of indoleamine 2,3-dioxygenase (IDO), an enzyme involved in tumor immunosuppression (Figure 2) [17,18]. In the last few years, the dithiocarbamate moiety has been incorporated into numerous cores such as benzoxazole [19], triazole [20], 2,4-diarylaminopyrimidine [21], quinazolinone [22,23], chalcone [24], pyridazinone [25] to develop new hybrid compounds displaying in vitro or in vivo antiproliferative activities.

Taking the abovementioned facts into account and that, to our knowledge, no anticancer activity data have been previously reported for compounds containing the phthalazinone and dithiocarbamate scaffolds in the same molecule, we designed a new series of phthalazinone-dithiocarbamate hybrids, compounds **6**–**9** (Figure 2). The target hybrids could be considered as phthalazinone analogues of brassinin (**5**), in which the indole nucleus was exchanged for a phthalazinone moiety with different substitution patterns at C4 and the S-methyl group was replaced by unsaturated fragments of different sizes, such as propargyl, benzyl or several *p*-substituted benzyl groups, some of them present in brassinin analogues and related compounds with potent antiproliferative activity [17,18]. In addition, since the N2 and C4 positions appear to be important for modulating the anticancer activity of the phthalazinone core [7,8,26], we have explored both locations to include the dithiocarbamate scaffold. Herein, we describe the synthesis, in vitro antiproliferative activity, and structure-activity relationships for these novel phthalazinone-dithiocarbamate hybrids **6**–**9**. SwissADME and ProTox-II programs were used to predict the pharmacokinetic properties and toxicity of some of the target compounds.

## 2. Results and Discussion

### 2.1. Chemistry

**Scheme 1:**
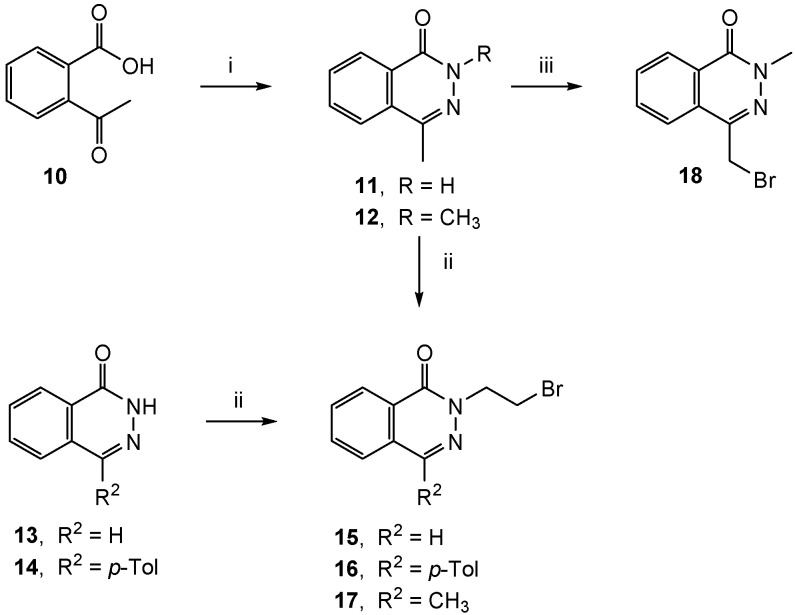
*Reagents and conditions*: (i) hydrazine hydrate or methyl hydrazine, ethanol reflux, 5 h, 91% (**11**), 98% (**12**); (ii) 1,2-dibromoethane, K_2_CO_3_, DMF 60 °C, 1.5 h, 50% (**15**), 75% (**16**), 45% (**17**); (iii) NBS, (BzO)_2_, acetonitrile, reflux, 12 h, 56% (**18**).

**Scheme 2:**
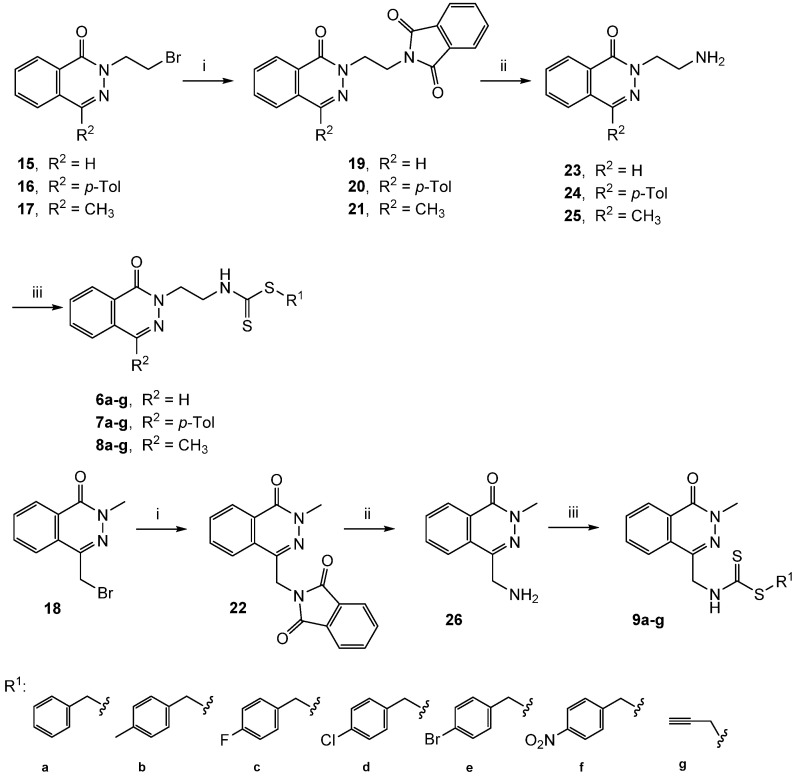
*Reagents and conditions*: (i) potassium phthalimide, DMF reflux, 8 h, 81% (**19**), 93% (**20**), 86% (**21**), 83% (**22**); (ii) hydrazine hydrate, ethanol reflux, 7 h, 2M HCl, 60 °C 2 h, 90 °C 1 h, Amberlyst A26 (OH), 97% (**23**), 80% (**24**), 99% (**25**), 94% (**26**); (iii) CS_2_, K_3_PO_4_, DMF, appropriate benzyl bromide or propargyl bromide, DMF, r. t., 3 h, 59% (**6a**), 70% (**6b**), 68% (**6c**), 67% (**6d**), 76% (**6e**), 91% (**6f**), 26% (**6g**), 55% (**7a**), 61% (**7b**), 80% (**7c**), 74% (**7d**), 64% (**7e**), 68% (**7f**), 54% (**7g**), 83% (**8a**), 61% (**8b**), 59% (**8c**), 78% (**8d**), 70% (**8e**), 59% (**8f**), 19% (**8g**), 71% (**9a**), 66% (**9b**), 22% (**9c**), 68% (**9d**), 85% (**9e**), 66% (**9f**), 36% (**9g**).

The proposed compounds were synthesized by functionalization of phthalazinone scaffolds **11**–**14** and via the corresponding alkyl bromides **15**–**18**, using the primary amines **23**–**26** as key intermediates, as illustrated in Scheme 1 and Scheme 2. Phthalazinones **11**–**12**, both substituted at C4 with a methyl group, were obtained in very good yields from 2-acetyl benzoic acid (**10**) by refluxing with hydrazine hydrate or methyl hydrazine in ethanol. On the other hand, unsubstituted phthalazinone **13** and its *p*-tolyl derivative **14** are commercially available.

The synthesis of 2-(2-bromoethyl)phthalazinones **15**–**17**, precursors of analogues **6**–**8**, was performed by N-alkylation of the adequate phthalazinone with an excess of 1,2-dibromoethane, in DMF at 60 °C, using K_2_CO_3_ as a base (Scheme 1). In this reaction, alkyl bromides **15**–**17** were the major products (45–75% yield), also obtaining as by-products the corresponding N,N′-dialkylation dimers. The 4-bromomethyl phthalazinone **18**, a precursor of the **9** analogues, was synthesized from **12** by Wohl–Ziegler’s bromination, with N-bromosuccinimide and benzoyl peroxide and using acetonitrile as an alternative solvent to carbon tetrachloride, to improve the substrate solubility (Scheme 1).

Synthesis of the key intermediates **23**–**26** was accomplished by adapting Gabriel’s method (Scheme 2). The reaction of bromoalkylphthalazinones **15**–**18** with potassium phthalimide provided the corresponding isoindolidine-1,3-diones in good yields, which were converted into the amines **23**–**26** by hydrazinolysis and acidification with 2M HCl followed by treatment with Amberlyst A26 (OH).

Finally, the phthalazinone-dithiocarbamate hybrids **6**–**9** were successfully obtained by a one-pot reaction, treating the adequate aminoalkyl phthalazinone with carbon disulfide, anhydrous H_3_PO_4_, and the different benzyl or propargyl bromides, in DMF, between 0 °C and room temperature (Scheme 2).

### 2.2. Pharmacology

The target compounds **6**–**9** were screened in vitro for their antiproliferative effects against three cell cancer lines, A2780 (human ovarian carcinoma), NCI-H460 (human lung carcinoma), and MCF-7 (human breast adenocarcinoma) through the 3-(4,5-dimethylthiazol-2-yl)-2,5-diphenyl-2H-tetrazolium bromide (MTT) assay and using cisplatin as a positive control [27]. The obtained results from this preliminary study are detailed in Table 1 and Table 2.

As can be observed, significant differences in antiproliferative effects were detected for the target compounds depending on the dithiocarbamate scaffold location. Thus, all compounds displaying the dithiocarbamate fragment at N2 (compounds **6**–**8**) were active against the A2780 cell line, with the IC_50_ values ranging from 5.20 to 88 µM, most of them were also active against the MCF-7 cell line, and only one of them displayed moderate activity toward the NCI-H460 cell line (compound **7g**, IC_50_ = 43 ± 1 µM).

However, the compounds **9**, containing the dithiocarbamate moiety at C4, exhibited moderate to good antiproliferative activity versus the three cancer cell lines studied. In addition, the selectivity displayed by almost all of them to the NCI-H460 cell line is noteworthy. Particularly, the analogues **9a**, **9b**, and **9d**, containing a benzyl, 4-methylbenzyl, or 4-chlorobenzyl group linked to the sulfur atom, respectively, were the most potent compounds against this lung cancer cell line, with IC_50_ values below 10 µM and close to that of cisplatin (IC_50_ = 5.54 ± 0.23 µM), the reference drug.

In the case of compounds **6**–**8** containing the dithiocarbamate scaffold at N2 and different substitution pattern at C4, regarding the A-2780 ovarian cancer cell line, the best results were achieved with the *p*-bromobenzyl derivatives **6e** and **8e**, and the propargyl derivative **6g**, with IC_50_ values of 5.53 ± 0.09, 7.51 ± 0.13 and 5.20 ± 0.13 µM, respectively.

In addition, the bromine atom substitution in compounds **6e** and **8e** (C4 methyl analogue) by other halogens, such as fluorine and chlorine, nitro or methyl groups, or even its removal, caused a decrease in activity. The replacement bromine-chlorine in the series of S-benzyl derivatives **6a**–**f** appeared to be the best tolerated, while a better result was achieved with the bromine-nitro substitution in the case of their C4 methyl analogues **8a**–**f**. This seems to indicate that the magnitude and electronegativity of the substituent in both series significantly affected the activity. However, no significant differences in the antiproliferative activity were observed within the series of S-benzyl analogues **7a**–**f** containing a *p*-tolyl group at C4, which displayed IC_50_ values between 10 and 20 µM.

Concerning to MCF-7 cell line, most of the S-benzyl analogues containing the dithiocarbamate fragment at N2 (compounds **6**–**8a**–**f**) exhibited moderate inhibitory activity against this cell line, specifically when the substituent at the *para* position was a bromine, chlorine, nitro or methyl group. However, the replacement of the benzyl moiety in compounds **6a**–**f** by a propargyl group significantly improved the antiproliferative activity against the MCF-7 cell line. Thus, compound **6g**, with an IC_50_ value of 7.64 ± 0.5, was 1.7 times more potent than cisplatin (IC_50_ = 13 ± 1) on the same cancer cell line.

The results obtained for compounds **6**–**8** revealed analogues **6e**, **8e**, and **6g** as the most interesting, compound **6g** being the most promising because of its potency and selectivity versus the A-2780 and MCF-7 cell lines.

On the other hand, as was previously mentioned, most of the analogues displaying the dithiocarbamate moiety at C4 (compounds **9**), were active toward the NCI-H460 cell line, these being **9a**, **9b**, and **9d**, with IC_50_ values of 7.36 ± 0.08, 8.49 ± 0.25 and 7.77 ± 0.17, respectively, the most potent and selective. Interestingly, the propargyl group of the **9** compounds also provided the best result against the A-2780 cell line, since the compound **9g**, with an IC_50_ value of 6.75 ± 0.12, was the most active derivative against this cell line.

### 2.3. Drug-Like and Toxicity Properties Prediction

To estimate the potential of target compounds as drugs in vivo, several physicochemical properties related to ADME (absorption, distribution, metabolism, and excretion) processes, pharmacokinetic and drug-likeness aspects, were calculated for the most active compounds (**6e**, **8e**, **6g**, **9a**–**b**, **9d**, and **9g**) using the SwissADME web tool [28]. Table 3 includes the computed values for simple physicochemical and lipophilicity descriptors, among them the *n*-octanol/water partition coefficient (log P_o/w_) and topological polar surface area (TPSA), two factors affecting the bioavailability.

As can be seen in Table 3, all the studied compounds showed good lipophilicity properties (consensus log P_o/w_ from 2.28 to 4.24). The TPSA value for most of them (**6e**, **8e**, **6g**, **9a**–**b**, and **9d**) was 104.31 Å^2^, that for compound **9g** was 92.28 Å^2^. Compounds with TPSA values ranging between 20 and 130 Å^2^ are predicted as favorable for passive diffusion across the gastrointestinal wall [28]. On the other hand, moderate polar molecules (TPSA < 79 Å^2^) and those that are acceptably lipophilic (log *P*_o/w_ from 0.4 to 6.0) have a high probability for crossing the blood–brain barrier (BBB) [29], which is not desirable when only peripheral effects are of interest.

**Figure 3:**
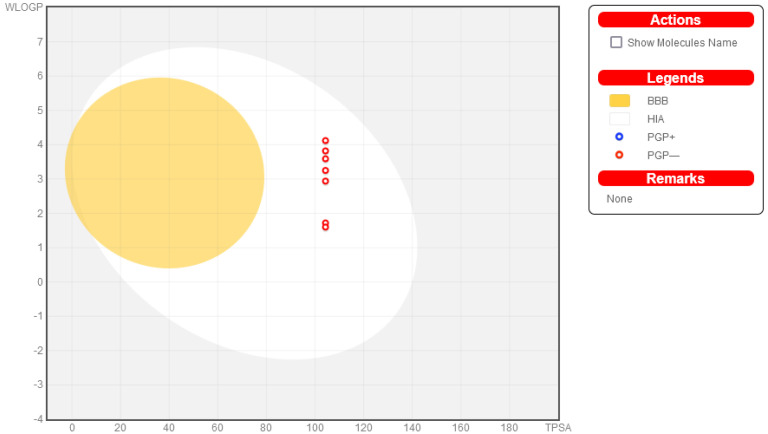
Predicted BOILED-egg model for compounds **6e**, **8e**, **6g**, **9a**–**b**, **9d**, and **9g**.

In connection to this, the brain or intestinal estimated permeation method (BOILED-egg), an accurate predictive model for the evaluation of human intestinal absorption (HIA) and brain access by computing lipophilicity and polarity, showed all compounds in the white area (Figure 3), the physicochemical space of molecules with a high probability of absorption after oral administration, and none of them in the space of molecules with high probability to cross the BBB (yellow area or yolk). The prediction reported an interesting pharmacokinetic profile for these compounds, regarding the oral absorption and the absence of effects on the central nervous system (CNS). Figure 3 also reveals that none of the seven studied compounds was a substrate for P-glycoprotein (PGP), with a key role in the active efflux of drugs across biological membranes, including the gastrointestinal wall [30]. In addition, PGP, due to its overexpression in some tumor cells, is related to multidrug resistance in cancers [31].

Regarding the interaction of these compounds with isoenzymes of the CYP450 superfamily, and in particular, with the five most significant isoforms (CYP1A2, CYP2C19, CYP2C9, CYP2D6, and CYP3A4) for drug metabolism, S-propargyl analogues **6g** and **9g** were predicted as inhibitors of CYP1A2, CYP2C19, and CYP2C9, while S-benzyl derivatives **6e**, **8e**, **9a**–**b** and **9d** were predicted as inhibitors of four (CYP1A2, CYP2C19, CYP2C9, and CYP3A4) of the five major CYP450 isoenzymes and, consequently, they would be more resistant to metabolism (Table S1).

**Figure 4:**
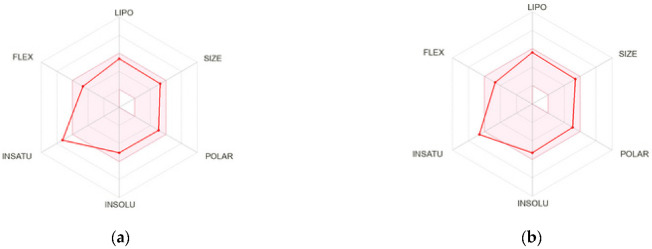

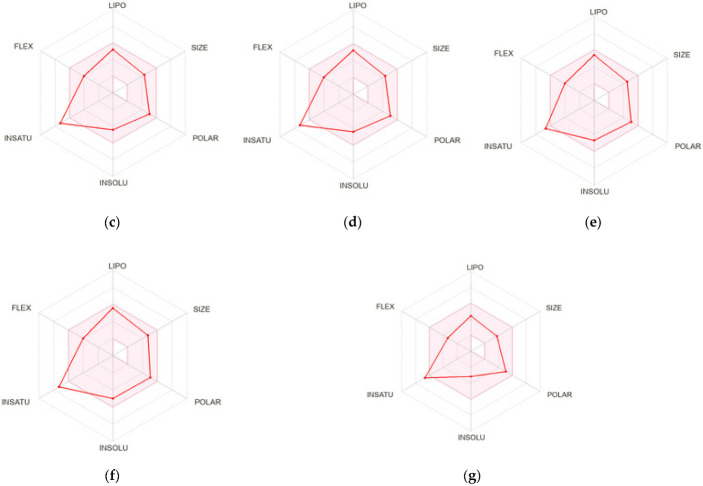
Bioavailability radar: (**a**) compound **6e**; (**b**) compound **8e**; (**c**) compound **6g**; (**d**) compound **9a**; (**e**) compound **9b**; (**f**) compound **9d**; (**g**) compound **9g**.

The bioavailability radar for the selected analogues, which includes the water solubility, unsaturation degree, flexibility, lipophilicity, size, and polarity parameters (Figure 4) showed that all compounds fell entirely in the pink area for five of the six physicochemical properties computed, all of them being outside of it for the unsaturation descriptor in this radar plot. The seven compounds fulfilled five of these six properties and passed the Lipinski [32], Ghose [33], Veber [34], Egan [35], and Muegge [36] filters associated with a good prediction of drug-likeness.

The toxicity of the seven selected compounds was calculated by using the ProTox-II server [37], a web platform which integrates 33 prediction models built from in vitro and in vivo assay data. This web server provides information about acute toxicity (DL_50_, mg/kg), hepatotoxicity, and several toxicity endpoints, including carcinogenicity, mutagenicity, immunotoxicity and cytotoxicity to human cells. In Table 4, are detailed the predicted DL_50_ values, toxicity category, as well as the percentages of molecular similarity and prediction accuracy.

The program establishes six toxicity categories, from the most to the least toxic, considering the DL_50_ thresholds. In this case, all compounds were classified as toxics of class 4 for acute oral toxicity (harmful if swallowed), with DL_50_ values of 350 mg/kg (**6e**, **8e**, and **6g**) or 500 mg/kg (**9a**–**b**, **9d**, and **9g**), considering an average of molecular similarity around 50% and a prediction accuracy higher than 50% (Table 4). In addition, all compounds were classified as lacking hepatotoxicity, immunotoxicity, mutagenicity and cytotoxicity, with probability values ranging from 0.51 to 0.99. However, four of them, specifically compounds **6g**, **9a**–**b**, and **9g** were predicted as potentially carcinogenic with a probability value of 0.53 (Table S2). Overall, three of the seven selected compounds (**6e**, **8e**, and **9d**) displayed favorable toxicological profiles in accordance with the ProTox-II webserver.

## 3. Materials and Methods

### 3.1. Materials and Instrumentation

Air-sensitive reactions were performed under an Ar atmosphere. Solvents were dried before use following standard procedures. Reactions were supervised by qualitative thin-layer chromatography (TLC) using silica gel plates (Merck 60 F254, 0.25 mm). Flash column chromatography was performed on silica gel Merck 60 (230–400 mesh) under pressure. ^1^H NMR, ^13^C NMR, and DEPT spectra were registered on Bruker DPX 400 and Bruker ARX 400 spectrometers, in CDCl_3_, CD_3_OD, or DMSO-*d*_6_ with TMS as an internal standard. Chemical shifts (δ) are given in parts per million (ppm) and coupling constants (*J*) in Hertz (Hz). High-resolution mass spectra (HRMS) were recorded on a VG Autospec M and Bruker FTMS APEX XIII spectrometers. Melting points were measured in a Stuart Scientific apparatus. Synthesis of phthalazinones **11**–**12** and 4-bromomethyl phthalazinone **18** was performed as previously described [38,39]. Synthesis of 2-(2-bromoethyl) phthalazinones **15**–**17**, which was accomplished by adapting procedures previously reported, as detailed in the Supplementary Materials Section.

### 3.2. Chemical Synthesis

#### 3.2.1. General Procedure for the Preparation of 2-(Phthalazinylalkyl)isoindoline-1,3-Diones (**19**–**22**)

To a solution of phthalazinone **15**–**18** (0.47 mmol) in DMF (7 mL) was added phthalimide potassium salt (0.62 mmol). The reaction mixture was stirred at reflux for 8 h and then at r.t. overnight. After the addition of H_2_O (10 mL), the white precipitate formed was filtered and dried in vacuum affording the desired compound.

*2-(2-(1-Oxophthalazin-2(1H)-yl)ethyl)isoindoline-1,3-dione* (**19**). White solid; yield: 81%; R*_f_* = 0.2 (hexane/EtOAc, 1:1); ^1^H NMR (CDCl_3_): *δ* = 8.35 (d, 1H, *J* = 7.8 Hz, H8 phthalazine), 7.98 (s, 1H, H4 phthalazine), 7.80–7.71 (m, 4H, Ar), 7.68–7.61 (m, 3H, Ar), 4.53 (t, 2H, *J* = 5.5 Hz, H2′), 4.18 (t, 2H, *J* = 5.5 Hz, H1′); ^13^C NMR (CDCl_3_): *δ* = 168.3 (CO), 159.8 (C1 phthalazine), 138.0 (C4 phthalazine), 134.0, 133.2, 132.1, 131.8, 129.7, 127.8, 126.8, 126.2, 123.4, 49.7 (C2′), 36.7 (C1′); HRMS (ESI): *m/z* [M+H]^+^ calcd for C_18_H_14_N_3_O_3_: 320.10297, found: 320.10340.

*2-(2-(1-Oxo-4-p-tolylphthalazin-2(1H)-yl)ethyl)isoindoline-1,3-dione* (**20**). White solid; yield: 93%; R*_f_* = 0.2 (hexane/EtOAc, 1:1); ^1^H NMR (CDCl_3_): *δ* = 8.46–8.42 (m, 1H, H8 phthalazine), 7.75–7.68 (m, 4H, Ar), 7.66–7.62 (m, 3H, Ar), 7.15–7.08 (m, 4H, Ar), 4.59 (t, 2H, *J* = 5.2 Hz, H2′), 4.22 (t, 2H, *J* = 5.2 Hz, H1′), 2.36 (s, 3H, CH_3_); ^13^C NMR (CDCl_3_): *δ* = 168.3 (CO), 159.6 (C1 phthalazine), 147.5, 138.9, 133.9, 132.9, 132.2, 132.1, 131.5, 129.3, 129.2, 129.1, 128.1, 127.2, 126.9, 123.3, 49.2 (C2′), 36.9 (C1′), 21.4 (CH_3_); HRMS (ESI): *m/z* [M+H]^+^ calcd for C_25_H_20_N_3_O_3_: 410.14992, found: 410.14979.

*2-(2-(4-Methyl-1-oxophthalazin-2(1H)-yl)ethyl)isoindoline-1,3-dione* (**21**). White solid; yield: 86%; R*_f_* = 0.2 (hexane/EtOAc, 1:1); ^1^H NMR (CDCl_3_): *δ* = 8.33 (d, 1H *J* = 7.6 Hz, H8 phthalazine), 7.75–7.70 (m, 3H, H5, H6, H7 phthalazine), 7.69–7.61 (m, 4H, Ar), 4.46 (t, 2H, *J* = 5.2 Hz, H2′), 4.13 (t, 2H, *J* = 5.2 Hz, H1′), 2.23 (s, 3H, CH_3_); ^13^C NMR (CDCl_3_): *δ* = 168.2 (CO), 159.6 (C1 phthalazine), 143.7 (C4 phthalazine), 133.8, 132.9, 132.1, 131.3, 129.7, 127.5, 127.0 (C8 phthalazine), 124.8, 123.1, 48.6 (C2′), 36.8 (C1′), 18.4 (CH_3_); HRMS (ESI): *m/z* [M+H]^+^ calcd for C_19_H_16_N_3_O_3_: 334.11862, found: 334.11899.

*2-((3-Methyl-4-oxo-3,4-dihydrophthalazin-1-yl)methyl)isoindoline-1,3-dione* (**22**). White solid; yield: 83%; R*_f_* = 0.4 (hexane/EtOAc, 1:2); ^1^H NMR (CDCl_3_): *δ* = 8.47 (d, 1H, *J* = 7.8 Hz, H5 phthalazine), 7.94–7.90 (m, 3H, H6, H7, H8 phthalazine), 7.87–7.76 (m, 4H, Ar), 5.19 (s, 2H, CH_2_), 3.68 (s, 3H, CH_3_); ^13^C NMR (CDCl_3_): *δ* = 168.2 (CO), 159.7 (C4 phthalazine), 138.6 (C1 phthalazine), 134.3, 133.2, 132.3, 131.8, 128.3, 128.0, 127.6 (C5 phthalazine), 123.7, 123.4, 39.8 (CH_3_), 38.6 (CH_2_); HRMS (ESI): *m/z* [M+H]^+^ calcd for C_18_H_14_N_3_O_3_: 320.10297, found: 320.10307.

#### 3.2.2. General Procedure for the Preparation of Aminoalkyl Phthalazin-1(2H)-Ones (**23**–**26**)

To a solution of compound **19**–**22** (0.74 mmol) in EtOH (11 mL) was added hydrazine hydrate (1.10 mmol) and the reaction mixture was refluxed for 7 h. The solvent was evaporated, 2M HCl (20.6 mmol) was added and the mixture was stirred for 2 h at 60 °C and then for 1 h at 90 °C, followed by the addition of H_2_O (11 mL) at r.t. The white precipitate formed was filtered off, washed with H_2_O and the solvent was removed under vacuum. The residue obtained was dissolved in MeOH (4 mL) and treated with Amberlyst A26 (OH) to afford the desired compound.

*2-(2-Aminoethyl)phthalazin-1(2H)-one* (**23**). Yellowish oil; yield: 97%; R*_f_* = 0.2 (CH_2_Cl_2_/MeOH/NH_3_, 90/9.5/0.5); ^1^H NMR (CD_3_OD): *δ* = 8.37 (s, 1H, H4), 8.33 (d, 1H, *J* = 7.9 Hz, H8), 7.93–7.83 (m, 3H, H5, H6, H7), 4.38 (t, 2H, *J* = 6.1 Hz, H1′), 3.29–3.13 (m, 2H, H2′); ^13^C NMR (CD_3_OD): *δ* = 161.6 (C1), 140.5 (C4), 134.8, 133.2, 131.2, 128.7, 127.9, 127.1, 52.6 (C1′), 40.7 (C2′); HRMS (ESI): *m/z* [M+H]^+^ calcd for C_10_H_12_N_3_O: 190.09749, found: 190.09747.

*2-(2-Aminoethyl)-4-p-tolylphthalazin-1(2H)-one* (**24**). Yellowish oil; yield: 80%; R*_f_* = 0.2 (CH_2_Cl_2_/MeOH/NH_3_, 90/9.5/0.5); ^1^H NMR (CD_3_OD): *δ* = 8.38–8.30 (m, 1H, H8), 7.80–7.72 (m, 2H, Ar), 7.70–7.63 (m, 1H, Ar), 7.40 (d, 2H, *J* = 8.0 Hz, Ar), 7.28 (d, 2H, *J* = 8.0 Hz, Ar), 4.32 (t, 2H, *J* = 6.2 Hz, H1′), 3.20–3.10 (m, 2H, H2′), 2.39 (s, 3H, CH_3_); ^13^C NMR (CD_3_OD): *δ* = 160.8 (C1), 148.9, 140.4, 134.3, 133.3, 132.7, 130.6, 130.5, 130.1, 129.0, 127.9, 127.6, 54.3 (C1′), 41.3 (C2′), 21.4 (CH_3_); HRMS (ESI): *m/z* [M+H]^+^ calcd for C_17_H_18_N_3_O: 280.14444, found: 280.14445.

*2-(2-Aminoethyl)-4-methylphthalazin-1(2H)-one* (**25**). Yellowish oil; yield: 99%; R*_f_* = 0.2 (CH_2_Cl_2_/MeOH/NH_3_, 90/9.5/0.5); ^1^H NMR (CD_3_OD): *δ* = 8.17 (d, 1H, *J* = 7.3 Hz, H8), 7.83–7.64 (m, 3H, H5, H6, H7), 4.27–4.11 (m, 2H, H1′), 3.15–2.95 (m, 2H, H2′), 2.47 (s, 3H, CH_3_); ^13^C NMR (CD_3_OD): *δ* =161.1 (C1), 146.0 (C4), 134.4, 132.7, 130.7, 128.2, 127.3, 126.3, 53.9 (C1′), 41.2 (C2′), 18.9 (CH_3_); HRMS (ESI): *m/z* [M+H]^+^ calcd for C_11_H_14_N_3_O: 204.11314, found: 204.11314.

*4-(Aminomethyl)-2-methylphthalazin-1(2H)-one* (**26**). Yellowish oil; yield: 94%; R*_f_* = 0.2 (CH_2_Cl_2_/MeOH/NH_3_, 90/9.5/0.5); ^1^H NMR (CD_3_OD): *δ* = 8.25–8.16 (m, 1H, H8), 7.88–7.79 (m, 2H, Ar), 7.78–7.70 (m, 1H, Ar), 4.12 (s, 2H, CH_2_), 3.75 (s, 3H, CH_3_); ^13^C NMR (CD_3_OD): *δ* = 160.8 (C1), 147.1 (C4), 134.4, 132.7, 129.4, 128.0, 127.3 (C8), 125.1, 42.7 (CH_2_), 39.7 (CH_3_); HRMS (ESI): *m/z* [M+H]^+^ calcd for C_10_H_12_N_3_O: 190.09749, found: 190.09746.

#### 3.2.3. General Procedure for the Preparation of Phthalazinyle Alkyl Dithiocarbamates (**6**–**9**)

To a solution of compound **23**–**26** (0.15 mmol) in DMF (1.5 mL) at 0 °C was added K_3_PO_4_ (0.30 mmol) and dropwise CS_2_ (0.37 mmol) and the reaction mixture was stirred for 1 h at the same temperature. A solution of the appropriate alkyl bromide (0.15 mmol) in DMF (0.4 mL) was added and stirring was continued for 3h, allowing the reaction mixture to gradually reach r.t. The solvent was evaporated and the residue was purified by column chromatography on silica gel (hexane/EtOAc, 4:1) affording the desired compound.

*Benzyl N-(2-(1-oxophthalazin-2(1H)-yl)ethyl)dithiocarbamate* (**6a**). White solid; yield: 59%; R*_f_* = 0.2 (hexane/EtOAc, 2:1); ^1^H NMR (CDCl_3_): *δ* = 8.55 (br s, 1H, NH), 8.41 (d, 1H, *J* = 7.8 Hz, H8 phthalazine), 8.19 (s, 1H, H4 phthalazine), 7.86–7.75 (m, 2H, H6, H7 phthalazine), 7.70 (d, 2H, *J* = 7.8 Hz, H5 phthalazine), 7.34 (d, 2H, *J* = 7.0 Hz, Ar), 7.29–7.21 (m, 3H, Ar), 4.57–4.53 (m, 2H, H2′), 4.49 (s, 2H, CH_2_S), 4.20–4.10 (m, 2H, H1′); ^13^C NMR (CDCl_3_): *δ* = 197.7 (CS), 161.0 (C1 phthalazine), 139.0 (C4 phthalazine), 136.4, 133.7, 132.2, 129.7, 129.2, 128.6, 127.6, 127.5, 126.8, 126.4, 50.2 (C2′), 48.2 (C1′), 39.8 (CH_2_S); HRMS (ESI): *m/z* [M+H]^+^ calcd for C_18_H_18_N_3_OS_2_: 356.08858, found: 356.08830.

*4-Methylbenzyl N-(2-(1-oxophthalazin-2(1H)-yl)ethyl)dithiocarbamate* (**6b**). White solid; yield: 70%; R*_f_* = 0.3 (hexane/EtOAc, 2:1); ^1^H NMR (CDCl_3_): *δ* = 8.50 (br s, 1H, NH), 8.41 (d, 1H, *J* = 7.7 Hz, H8 phthalazine), 8.19 (s, 1H, H4 phthalazine), 7.87–7.76 (m, 2H, H6, H7 phthalazine), 7.72 (d, 1H, *J* = 7.7 Hz, H5 phthalazine), 7.23 (d, 2H, *J* = 7.8 Hz, Ar), 7.07 (d, 2H, *J* = 7.8 Hz, Ar), 4.59–4.53 (m, 2H, H2′), 4.45 (s, 2H, CH_2_S), 4.19–4.11 (m, 2H, H1′), 2.30 (s, 3H, CH_3_); ^13^C NMR (CDCl_3_): *δ* = 197.9 (CS), 161.0 (C1 phthalazine), 139.1 (C4 phthalazine), 137.2, 133.7, 133.2, 132.2, 129.7, 129.3, 129.1, 127.6, 126.8 (C8 phthalazine), 126.4 (C5 phthalazine), 50.2 (C2′), 48.3 (C1′), 39.6 (CH_2_S), 21.2(CH_3_); HRMS (ESI): *m/z* [M+H]^+^ calcd for C_19_H_20_N_3_OS_2_: 370.10423, found: 370.10389.

*4-Fluorobenzyl N-(2-(1-oxophthalazin-2(1H)-yl)ethyl) dithiocarbamate* (**6c**). White solid; yield: 68%; R*_f_* = 0.2(hexane/EtOAc, 2:1); ^1^H NMR (CDCl_3_): *δ* = 8.55 (br s, 1H, NH), 8.41 (d, 2H, *J* = 7.5 Hz, H8 phthalazine), 8.20 (s, 1H, H4 phthalazine), 7.88–7.75 (m, 2H, H6, H7 phthalazine), 7.72 (d, 2H, *J* = 7.5 Hz, H5 phthalazine), 7.35–7.27 (m, 2H, Ar), 6.97–6.90 (m, 2H, CHCF), 4.59–4.53 (m, 2H, H2′), 4.46 (s, 2H, CH_2_S), 4.21–4.08 (m, 2H, H1′); ^13^C NMR (CDCl_3_): *δ* = 197.5 (CS), 163.4 (CF), 161.1 (C1 phthalazine), 139.1 (C4 phthalazine), 133.8, 132.5, 132.3, 130.8, 130.7, 129.8, 127.6, 126.9 (C8 phthalazine), 126.4 (C5 phthalazine), 115.6 (*C*HCF), 115.4 (*C*HCF), 50.3 (C2′), 48.5 (C1′), 39.0 (CH_2_S); HRMS (ESI): *m/z* [M+H]^+^ calcd for C_18_H_17_FN_3_OS_2_: 374.07916, found: 374.07859.

*4-Chlorobenzyl N-(2-(1-oxophthalazin-2(1H)-yl)ethyl)dithiocarbamate* (**6d**). White solid; yield: 67%; R*_f_* = 0.2 (hexane/EtOAc, 2:1); ^1^H NMR (CDCl_3_): *δ* = 8.55 (br s, 1H, NH), 8.42 (d, 1H, *J* = 7.8 Hz, H8 phthalazine), 8.20 (s, 1H, H4 phthalazine), 7.88–7.78 (m, 2H, H6, H7 phthalazine), 7.73 (d, 1H, *J* = 7.8 Hz, H5 phthalazine), 7.28 (d, 2H, *J* = 8.4 Hz, Ar), 7.22 (d, 2H, *J* = 8.4 Hz, Ar), 4.59–4.54 (m, 2H, H2′), 4.46 (s, 2H, CH_2_S), 4.18–4.10 (m, 2H, H1′); ^13^C NMR (CDCl_3_): *δ* = 197.3 (CS), 161.1 (C1 phthalazine), 139.1 (C4 phthalazine), 135.4, 133.8, 133.3, 132.3, 130.5, 129.7, 128.8, 127.6, 126.9, 126.4, 50.3 (C2′), 48.6 (C1′), 39.0 (CH_2_S); HRMS (ESI): *m/z* [M+H]^+^ calcd for C_18_H_17_ClN_3_OS_2_: 390.04961, found: 390.04983.

*4-Bromobenzyl N-(2-(1-oxophthalazin-2(1H)-yl)ethyl)dithiocarbamate* (**6e**). White solid; yield: 76%; R*_f_* = 0.2 (hexane/EtOAc, 2:1); ^1^H NMR (CDCl_3_): *δ* = 8.57 (br s, 1H, NH), 8.40 (d, 1H, *J* = 7.7 Hz, H8 phthalazine), 8.19 (s, 1H, H4 phthalazine), 7.88–7.76 (m, 2H, H6, H7 phthalazine), 7.71 (d, 1H, *J* = 7.7 Hz, H5 phthalazine), 7.36 (d, 2H, *J* = 8.2 Hz, Ar), 7.21 (d, 2H, *J* = 8.2 Hz, Ar), 4.58–4.52 (m, 2H, H2′), 4.44 (s, 2H, CH_2_S), 4.20–4.10 (m, 2H, H1′); ^13^C NMR (CDCl_3_): *δ* = 197.2 (CS), 161.1 (C1 phthalazine), 139.1 (C4 phthalazine), 136.0, 133.8, 132.3, 131.7, 130.9, 129.7, 127.6, 126.9 (C8 phthalazine), 126.4 (C5 phthalazine), 121.4 (CBr), 50.3 (C2′), 48.5 (C1′), 39.0 (CH_2_S); HRMS (ESI): *m/z* [M+H]^+^ calcd for C_18_H_17_BrN_3_OS_2_: 433.99909, found: 433.99854.

*4-Nitrobenzyl N-(2-(1-oxophthalazin-2(1H)-yl)ethyl) dithiocarbamate* (**6f**). White solid; yield: 91%; R*_f_* = 0.1 (hexane/EtOAc, 2:1); ^1^H NMR (CDCl_3_): *δ* = 8.66 (br s, 1H, NH), 8.45 (d, 2H, *J* = 7.8 Hz, H8 phthalazine), 8.24 (s, 1H, H4 phthalazine), 8.13 (d, 2H, *J* = 8.6 Hz, CHCNO_2_), 7.91–7.82 (m, 2H, H6, H7 phthalazine), 7.76 (d, 1H, *J* = 7.8 Hz, H5 phthalazine), 7.54 (d, 2H, *J* = 8.6 Hz, Ar), 4.62 (s, 2H, CH_2_S), 4.61–4.57 (m, 2H, H2′), 4.21–4.14 (m, 2H, H1′); ^13^C NMR (DMSO): *δ* = 195.9 (CS), 158.7 (C1 phthalazine), 146.4, 146.1, 137.9 (C4 phthalazine), 133.4, 131.9, 129.9, 129.4, 127.1, 126.7, 125.7 (C8 phthalazine), 123.4 (*C*HCNO_2_), 48.8 (C2′), 44.8 (C1′), 37.1 (CH_2_S); HRMS (ESI): *m/z* [M+H]^+^ calcd for C_18_H_17_N_4_O_3_S_2_: 401.07366, found: 401.07363.

*Prop-2-ynyl N-(2-(1-oxophthalazin-2(1H)-yl)ethyl) dithiocarbamate* (**6g**). White solid; yield: 26%; R*_f_* = 0.2 (hexane/EtOAc, 2:1); ^1^H NMR (CDCl_3_): *δ* = 8.61 (br s, 1H, NH), 8.45 (d, 2H, *J* = 7.6 Hz, H8 phthalazine), 8.23 (s, 1H, H4 phthalazine), 7.88–7.80 (m, 2H, H6, H7 phthalazine), 7.74 (d, 2H, *J* = 7.6 Hz, H5 phthalazine), 4.60–4.54 (m, 2H, H2′), 4.19–4.12 (m, 2H, H1′), 4.00 (d, 2H, *J* = 2.6 Hz, CH_2_S), 2.18 (t, 1H, *J* = 2.6 Hz, HC≡); ^13^C NMR (CDCl_3_): *δ* = 195.8 (CS), 161.2 (C1 phthalazine), 139.2 (C4 phthalazine), 133.9, 132.3, 129.8, 127.7, 127.0 (C8 phthalazine), 126.4 (C5 phthalazine), 78.6 (C≡), 71.8 (HC≡), 50.3 (C2′), 48.7 (C1′), 24.0 (CH_2_S); HRMS (ESI): *m/z* [M+H]^+^ calcd for C_14_H_14_N_3_OS_2_: 304.05728, found: 304.05715.

*Benzyl N-(2-(1-oxo-4-p-tolylphthalazin-2(1H)-yl)ethyl)dithiocarbamate* (**7a**). White solid; yield: 55%; R*_f_* = 0.3(hexane/EtOAc, 2:1); ^1^H NMR (CDCl_3_): *δ* = 8.70 (br s, 1H, NH), 8.54–8.50 (m, 1H, H8 phthalazine), 7.84–7.74 (m, 3H, H5, H6, H7 phthalazine), 7.48 (d, *J* = 8.0 Hz, 2H, Ar), 7.36–7.31 (m, 4H, Ar), 7.28–7.22 (m, 3H, Ar), 4.68–4.60 (m, 2H, H2′), 4.45 (s, 2H, CH_2_S), 4.22–4.14 (m, 2H, H1′), 2.44 (s, 3H, CH_3_); ^13^C NMR (CDCl_3_): *δ* = 197.5 (CS), 160.6 (C1 phthalazine), 148.5, 139.6, 136.2, 133.3, 131.8, 131.7, 129.4, 129.3, 129.1, 128.5, 127.9, 127.4, 127.2, 127.1, 50.1 (C2′), 48.6 (C1′), 39.7 (CH_2_S), 21.4 (CH_3_); HRMS (ESI): *m/z* [M+H]^+^ calcd for C_25_H_24_N_3_OS_2_: 446.13553, found: 446.13492.

*4-Methylbenzyl N-(2-(1-oxo-4-p-tolylphthalazin-2(1H)-yl)ethyl)dithiocarbamate* (**7b**). White solid; yield: 61%; R*_f_* = 0.3 (hexane/EtOAc, 2:1); ^1^H NMR (CDCl_3_): *δ* = 8.70 (br s, 1H, NH), 8.53–8.49 (m, 1H, H8 phthalazine), 7.83–7.77 (m, 3H, H5, H6, H7 phthalazine), 7.48 (d, 2H, *J* = 7.9 Hz, Ar), 7.33 (d, 2H, *J* = 7.9 Hz, Ar), 7.22 (d, 2H, *J* = 7.9 Hz, Ar), 7.07 (d, 2H, *J* = 7.9 Hz, Ar), 4.65–4.60 (m, 2H, H2′), 4.41 (s, 2H, CH_2_S), 4.19–4.14 (m, 2H, H1′), 2.44 (s, 3H, CH_3_), 2.30 (s, 3H, CH_3_); ^13^C NMR (CDCl_3_): *δ* = 197.7 (CS), 160.7 (C1 phthalazine), 148.6, 139.6, 137.2, 133.4, 133.1, 131.9, 131.8, 129.5, 129.4, 129.3, 129.3, 129.1, 128.0, 127.3, 127.2, 50.1 (C2′), 48.6 (C1′), 39.5 (CH_2_S), 21.5 (CH_3_), 21.2 (CH_3_); HRMS (ESI): *m/z* [M+H]^+^ calcd for C_26_H_26_N_3_OS_2_: 460.15118, found: 460.15043.

*4-Fluorobenzyl N-(2-(1-oxo-4-p-tolylphthalazin-2(1H)-yl)ethyl)dithiocarbamate* (**7c**). White solid; yield: 80%; R*_f_* = 0.2 (hexane/EtOAc, 2:1); ^1^H NMR (CDCl_3_): *δ* = 8.74 (br s, 1H, NH), 8.54–8.49 (m, 1H, H8 phthalazine), 7.83–7.76 (m, 3H, H5, H6, H7 phthalazine), 7.48 (d, 2H, *J* = 7.9 Hz, Ar), 7.35–7.27 (m, 4H, Ar), 6.98–6.90 (m, 2H, CHCF), 4.66–4.59 (m, 2H, H2′), 4.44 (s, 2H, CH_2_S), 4.22–4.13 (m, 2H, H1′), 2.45 (s, 3H, CH_3_); ^13^C NMR (CDCl_3_): *δ* = 197.3 (CS), 163.4 (CF), 160.7 (C1 phthalazine), 148.6, 139.7, 133.4, 131.9, 131.8, 130.8, 130.8, 129.5, 129.4, 128.0, 127.3, 127.2, 115.6 (*C*HCF), 115.4 (*C*HCF), 50.2 (C2′), 48.7 (C1′), 38.9 (CH_2_S), 21.5 (CH_3_); HRMS (ESI): *m/z* [M+H]^+^ calcd for C_25_H_23_FN_3_OS_2_: 464.12611, found: 464.12534.

*4-Chlorobenzyl N-(2-(1-oxo-4-p-tolylphthalazin-2(1H)-yl)ethyl)dithiocarbamate* (**7d**). White solid; yield: 74%; R*_f_* = 0.2(hexane/EtOAc, 2:1); ^1^H NMR (CDCl_3_): *δ* = 8.75 (br s, 1H, NH), 8.53–8.49 (m, 1H, H8 phthalazine), 7.83–7.77 (m, 3H, H5, H6, H7 phthalazine), 7.47 (d, 2H, *J* = 8.0 Hz, Ar), 7.33 (d, 2H, *J* = 8.0 Hz, Ar), 7.27 (d, 2H, *J* = 8.4 Hz, Ar), 7.21 (d, 2H, *J* = 8.4 Hz, Ar), 4.65–4.60 (m, 2H, H2′), 4.43 (s, 2H, CH_2_S), 4.21–4.13 (m, 2H, H1′), 2.45 (s, 3H, CH_3_); ^13^C NMR (CDCl_3_): *δ* = 197.0 (CS), 160.7 (C1 phthalazine), 148.6, 139.7, 135.3, 133.4, 133.2, 131.9, 131.8, 130.5, 129.5, 129.4, 129.3, 128.7, 127.9, 127.3, 127.2, 50.2 (C2′), 48.7 (C1′), 38.9 (CH_2_S), 21.5 (CH_3_); HRMS (ESI): *m/z* [M+H]^+^ calcd for C_25_H_23_ClN_3_OS_2_: 480.09656, found: 480.09582.

*4-Bromobenzyl N-(2-(1-oxo-4-p-tolylphthalazin-2(1H)-yl)ethyl)dithiocarbamate* (**7e**). White solid; yield: 64%; R*_f_* = 0.3 (hexane/EtOAc, 2:1); ^1^H NMR (CDCl_3_): *δ* = 8.75 (br s, 1H, NH), 8.52–8.49 (m, 1H, H8 phthalazine), 7.81–7.76 (m, 3H, H5, H6, H7 phthalazine), 7.47 (d, 2H, *J* = 7.9 Hz, Ar), 7.38–7.32 (m, 4H, Ar), 7.20 (d, 2H, *J* = 8.2 Hz, Ar), 4.65–4.59 (m, 2H, H2′), 4.41 (s, 2H, CH_2_S), 4.20–4.12 (m, 2H, H1′), 2.45 (s, 3H, CH_3_); ^13^C NMR (CDCl_3_): *δ* = 197.0 (CS), 160.7 (C1 phthalazine), 148.6, 139.7, 135.9, 133.4, 131.9, 131.8, 131.7, 130.9, 129.5, 129.4, 128.0, 127.3, 127.2, 121.3 (CBr), 50.2 (C2′), 48.8 (C1′), 38.9 (CH_2_S), 21.5 (CH_3_); HRMS (ESI): *m/z* [M+H]^+^ calcd for C_25_H_23_BrN_3_OS_2_: 524.04604, found: 524.04536.

*4-Nitrobenzyl N-(2-(1-oxo-4-p-tolylphthalazin-2(1H)-yl)ethyl)dithiocarbamate* (**7f**). White solid; yield: 68%; R*_f_* = 0.2 (hexane/EtOAc, 2:1); ^1^H NMR (CDCl_3_, δ): 8.84 (br s, 1H, NH), 8.52–8.48 (m, 1H, H8 phthalazine), 8.08 (d, 2H, *J* = 8.8 Hz, CHCNO_2_), 7.83–7.77 (m, 3H, H5, H6, H7 phthalazine), 7.52–7.45 (m, 4H, Ar), 7.33 (d, 2H, *J* = 7.8 Hz, Ar), 4.64–4.61 (m, 2H, H2′), 4.58 (s, 2H, CH_2_S), 4.21–4.13 (m, 2H, H1′), 2.45 (s, 3H, CH_3_); ^13^C NMR (CDCl_3_, δ): 196.2 (CS), 160.9 (C1 phthalazine), 148.7, 147.2, 145.2, 139.7, 133.5, 131.9, 131.8, 130.0, 129.5, 129.4, 127.9, 127.3, 127.2, 123.7 (*C*HCNO_2_), 50.4 (C2′), 49.1 (C1′), 38.7 (CH_2_S), 21.5 (CH_3_); HRMS (ESI): *m/z* [M+H]^+^ calcd for C_25_H_23_N_4_O_3_S_2_: 491.12061, found: 491.12036.

*Prop-2-ynyl N-(2-(1-oxo-4-p-tolylphthalazin-2(1H)-yl)ethyl)dithiocarbamate* (**7g**). White solid; yield: 54%; R*_f_* = 0.2 (hexane/EtOAc, 2:1); ^1^H NMR (CDCl_3_): *δ* = 8.80 (br s, 1H, NH), 8.53 (d, 2H, *J* = 7.4 Hz, H8 phthalazine), 7.84–7.77 (m, 3H, H5, H6, H7 phthalazine), 7.48 (d, 2H, *J* = 7.9 Hz, Ar), 7.35 (d, 2H, *J* = 7.9 Hz, Ar), 4.66–4.61 (m, 2H, H2′), 4.21–4.13 (m, 2H, H1′), 3.97 (d, 2H, *J* = 2.6 Hz, CH_2_S), 2.46 (s, 3H, CH_3_), 2.18 (t, 1H, *J* = 2.6 Hz, HC≡); ^13^C NMR (CDCl_3_): *δ* = 195.6 (CS), 160.8 (C1 phthalazine), 148.7, 139.7, 133.5, 131.9, 131.9, 129.6, 129.4, 128.0, 127.4, 127.2, 78.6 (C≡), 71.8 (HC≡), 50.2 (C2′), 48.9 (C1′), 24.0 (CH_2_S), 21.5 (CH_3_); HRMS (ESI): *m/z* [M+H]^+^ calcd for C_21_H_20_N_3_OS_2_: 394.10423, found: 394.10354.

*Benzyl N-(2-(4-methyl-1-oxophthalazin-2(1H)-yl)ethyl)dithiocarbamate* (**8a**). White solid; yield: 83%; R*_f_* = 0.2 (hexane/EtOAc, 4:1); ^1^H NMR (CDCl_3_): *δ* = 8.73 (br s, 1H, NH), 8.44 (d, 2H, *J* = 8.3 Hz, H8 phthalazine), 7.87–7.81 (m, 1H, Ar), 7.81–7.74 (m, 2H, Ar), 7.34 (d, 2H, *J* = 7.3 Hz, Ar), 7.29–7.21 (m, 3H, Ar), 4.55–4.50 (m, 2H, H2′), 4.49 (s, 2H, CH_2_S), 4.17–4.09 (m, 2H, H1′), 2.59 (s, 3H, CH_3_); ^13^C NMR (CDCl_3_): *δ* = 197.6 (CS), 161.0 (C1 phthalazine), 145.1 (C4 phthalazine), 136.5, 133.5, 131.8, 129.9, 129.2, 128.6, 127.5, 127.4, 127.3, 125.1, 49.9 (C2′), 48.8 (C1′), 39.8 (CH_2_S), 18.9 (CH_3_); HRMS (ESI): *m/z* [M+H]^+^ calcd for C_19_H_20_N_3_OS_2_: 370.10423, found: 370.10393.

*4-Methylbenzyl N-(2-(4-methyl-1-oxophthalazin-2(1H)-yl)ethyl)dithiocarbamate* (**8b**). White solid; yield: 61%; R*_f_* = 0.2 (hexane/EtOAc, 2:1); ^1^H NMR (CDCl_3_): *δ* = 8.71 (br s, 1H, NH), 8.46–8.42 (m, 1H, H8 phthalazine), 7.88–7.82 (m, 1H, Ar), 7.81–7.76 (m, 2H, Ar), 7.23 (d, 2H, *J* = 7.8 Hz, Ar), 7.07 (d, 2H, *J* = 7.8 Hz, Ar), 4.54–4.51 (m, 2H, H2′), 4.44 (s, 2H, CH_2_S), 4.16–4.09 (m, 2H, H1′), 2.59 (s, 3H, CH_3_C = N), 2.30 (s, 3H, CH_3_); ^13^C NMR (CDCl_3_): *δ* = 197.7 (CS), 161.0 (C1 phthalazine), 145.1 (C4 phthalazine), 137.2, 133.5, 133.2, 131.8, 129.9, 129.3, 129.1, 127.4, 127.2 (C8 phthalazine), 125.1, 49.9 (C2′), 48.7 (C1′), 39.5 (CH_2_S), 21.2 (CH_3_), 18.9 (CH_3_C = N); HRMS (ESI): *m/z* [M+H]^+^ calcd for C_20_H_22_N_3_OS_2_: 384.11988, found: 384.11941.

*4-Fluorobenzyl N-(2-(4-methyl-1-oxophthalazin-2(1H)-yl)ethyl)dithiocarbamate* (**8c**). White solid; yield: 59%; R*_f_* = 0.3 (hexane/EtOAc, 2:1); ^1^H NMR (CDCl_3_): *δ* = 8.78 (br s, 1H, NH), 8.45–8.41 (m, 1H, H8 phthalazine), 7.89–7.83 (m, 1H, Ar), 7.81–7.76 (m, 2H, Ar), 7.33–7.29 (m, 2H, Ar), 6.96–6.91 (m, 2H, CHCF), 4.54–4.51 (m, 2H, H2′), 4.46 (s, 2H, CH_2_S), 4.15–4.08 (m, 2H, H1′), 2.59 (s, 3H, CH_3_); ^13^C NMR (CDCl_3_): *δ* = 197.3 (CS), 163.4 (CF), 161.1 (C1 phthalazine), 145.2 (C4 phthalazine), 133.6, 132.5, 131.9, 130.8, 130.7, 129.9, 127.4, 127.3, 125.1, 115.6 (*C*HCF), 115.4 (*C*HCF), 50.0 (C2′), 48.9 (C1′), 38.9 (CH_2_S), 18.9 (CH_3_); HRMS (ESI): *m/z* [M+H]^+^ calcd for C_19_H_19_FN_3_OS_2_: 388.09481, found: 388.09433.

*4-Chlorobenzyl N-(2-(4-methyl-1-oxophthalazin-2(1H)-yl)ethyl)dithiocarbamate* (**8d**). White solid; yield: 78%; R*_f_* = 0.2 (hexane/EtOAc, 2:1); ^1^H NMR (CDCl_3_): *δ* = 8.77 (br s, 1H, NH), 8.42 (d, 2H, *J* = 7.5 Hz, H8 phthalazine), 7.86–7.81 (m, 1H, Ar), 7.80–7.73 (m, 2H, Ar), 7.27 (d, 2H, *J* = 8.2 Hz, Ar), 7.20 (d, 2H, *J* = 8.2 Hz, Ar), 4.53–4.49 (m, 2H, H2′), 4.45 (s, 2H, CH_2_S), 4.14–4.08 (m, 2H, H1′), 2.58 (s, 3H, CH_3_); ^13^C NMR (CDCl_3_): *δ* = 197.0 (CS), 161.1 (C1 phthalazine), 145.2 (C4 phthalazine), 135.4, 133.6, 133.2, 131.8, 130.5, 129.9, 128.7, 127.3, 127.2, 125.1, 50.0 (C2′), 48.9 (C1′), 38.9 (CH_2_S), 18.9 (CH_3_); HRMS (ESI): *m/z* [M+H]^+^ calcd for C_19_H_19_ClN_3_OS_2_: 404.06526, found: 404.06483.

*4-Bromobenzyl N-(2-(4-methyl-1-oxophthalazin-2(1H)-yl)ethyl)dithiocarbamate* (**8e**). White solid; yield: 70%; R*_f_* = 0.2 (hexane/EtOAc, 2:1); ^1^H NMR (CDCl_3_): *δ* = 8.76 (br s, 1H, NH), 8.46–8.43 (m, 1H, H8 phthalazine), 7.88–7.83 (m, 1H, Ar), 7.82–7.77 (m, 2H, Ar), 7.37 (d, 2H, *J* = 8.2 Hz, Ar), 7.22 (d, 2H, *J* = 8.2 Hz, Ar), 4.54–4.50 (m, 2H, H2′), 4.44 (s, 2H, CH_2_S), 4.15–4.08 (m, 2H, H1′), 2.60 (s, 3H, CH_3_); ^13^C NMR (CDCl_3_): *δ* = 197.0 (CS), 161.1 (C1 phthalazine), 145.2 (C4 phthalazine), 136.0, 133.6, 131.9, 131.7, 130.9, 129.9, 127.5, 127.3 (C8 phthalazine), 125.1, 121.4 (CBr), 50.0 (C2′), 49.0 (C1′), 39.0 (CH_2_S), 19.0 (CH_3_); HRMS (ESI): *m/z* [M+H]^+^ calcd for C_19_H_19_BrN_3_OS_2_: 448.01474, found: 448.01421.

*4-Nitrobenzyl N-(2-(4-methyl-1-oxophthalazin-2(1H)-yl)ethyl)dithiocarbamate* (**8f**). White solid; yield: 59%; R*_f_* = 0.1 (hexane/EtOAc, 2:1); ^1^H NMR (CDCl_3_): *δ* = 8.90 (br s, 1H, NH), 8.46–8.41 (m, 1H, H8 phthalazine), 8.09 (d, 2H, *J* = 8.7 Hz, CHCNO_2_), 7.89–7.84 (m, 1H, Ar), 7.82–7.77 (m, 2H, Ar), 7.51 (d, 2H, *J* = 8.7 Hz, Ar), 4.58 (s, 2H, CH_2_S), 4.55–4.50 (m, 2H, H2′), 4.15–4.07 (m, 2H, H1′), 2.59 (s, 3H, CH_3_); ^13^C NMR (CDCl_3_): *δ* = 196.1 (CS), 161.2 (C1 phthalazine), 147.2 (CNO_2_), 145.3, 145.2, 133.7, 131.9, 130.0, 129.9, 127.4, 127.2, 125.2, 123.8 (*C*HCNO_2_), 50.2 (C2′), 49.3 (C1′), 38.7 (CH_2_S), 19.0 (CH_3_); HRMS (ESI): *m/z* [M+H]^+^ calcd for C_19_H_19_N_4_O_3_S_2_: 415.08931, found: 415.08914.

*Prop-2-ynyl N-(2-(4-methyl-1-oxophthalazin-2(1H)-yl)ethyl)dithiocarbamate* (**8g**). White solid; yield: 19%; R*_f_* = 0.2 (hexane/EtOAc, 2:1); ^1^H NMR (CDCl_3_): *δ* = 8.81 (br s, 1H, NH), 8.49–8.45 (m, 1H, H8 phthalazine), 7.89–7.78 (m, 3H, H5, H6, H7 phthalazine), 4.57–4.52 (m, 2H, H2′), 4.16–4.10 (m, 2H, H1′), 3.99 (d, 2H, *J* = 2.6 Hz, CH_2_S), 2.61 (s, 3H, CH_3_), 2.18 (t, 2H, *J* = 2.6 Hz, HC≡); ^13^C NMR (CDCl_3_): *δ* = 195.6 (CS), 161.2 (C1 phthalazine), 145.3 (C4 phthalazine), 133.6, 131.9, 130.0, 127.5, 127.4 (C8 phthalazine), 125.1, 78.6 (C≡), 71.8 (HC≡), 50.0 (C2′), 49.1 (C1′), 24.0 (CH_2_S), 19.0 (CH_3_); HRMS (ESI): *m/z* [M+H]^+^ calcd for C_15_H_16_N_3_OS_2_: 318.07293, found: 318.07273.

*Benzyl N-((3-methyl-4-oxo-3,4-dihydrophthalazin-1-yl)methyl)dithiocarbamate* (**9a**). White solid; yield: 71%; R*_f_* = 0.3 (hexane/EtOAc, 2:1); ^1^H NMR (CDCl_3_): *δ* = 8.42–8.31 (m, 2H, NH, H5 phthalazine), 7.83–7.77 (m, 2H, Ar), 7.77–7.68 (m, 1H, Ar), 7.41 (d, 2H, *J* = 7.2 Hz, Ar), 7.34–7.26 (m, 3H, Ar), 5.19 (d, 2H, *J* = 4.2 Hz, CH_2_), 4.62 (s, 2H, CH_2_S), 3.76 (s, 3H, CH_3_); ^13^C NMR (CDCl_3_): *δ* = 197.8 (CS), 159.5 (C4 phthalazine), 139.9, 136.5, 133.6, 132.2, 129.2, 128.8, 128.0, 127.7, 127.6, 127.4, 123.7, 47.5 (CH_2_), 40.2 (CH_2_S), 39.5 (CH_3_); HRMS (ESI): *m/z* [M+H]^+^ calcd for C_18_H_18_N_3_OS_2_: 356.08858, found: 356.08825.

*4-Methylbenzyl N-((3-methyl-4-oxo-3,4-dihydrophthalazin-1-yl)methyl)dithiocarbamate* (**9b**). White solid; yield: 66%; R*_f_* = 0.2 (hexane/EtOAc, 2:1); ^1^H NMR (CDCl_3_): *δ* = 8.45 (br s, 1H, NH), 8.32 (d, 2H, *J* = 7.9 Hz, H5 phthalazine), 7.80–7.77 (m, 2H, Ar), 7.74–7.68 (m, 1H, Ar), 7.29 (d, 2H, *J* = 7.9 Hz, Ar), 7.11 (d, 2H, *J* = 7.9 Hz, Ar), 5.18 (d, 2H, *J* = 4.3 Hz, CH_2_), 4.57 (s, 2H, CH_2_S), 3.72 (s, 3H, CH_3_N), 2.32 (s, 3H, CH_3_); ^13^C NMR (CDCl_3_): *δ* = 197.9 (CS), 159.5 (C4 phthalazine), 140.1, 137.4, 133.6, 133.3, 132.2, 129.4, 129.1, 128.0, 127.5, 127.3, 123.7, 47.5 (CH_2_), 39.9 (CH_2_S), 39.5 (CH_3_N), 21.3 (CH_3_); HRMS (ESI): *m/z* [M+H]^+^ calcd for C_19_H_20_N_3_OS_2_: 370.10423, found: 370.10397.

*4-Fluorobenzyl N-((3-methyl-4-oxo-3,4-dihydrophthalazin-1-yl)methyl)dithiocarbamate* (**9c**). White solid; yield: 22%; R*_f_* = 0.2 (hexane/EtOAc, 2:1); ^1^H NMR (CDCl_3_): *δ* = 8.46 (br s, 1H, NH), 8.33 (d, 1H, *J* = 8.0 Hz, H5 phthalazine), 7.82–7.77 (m, 2H, Ar), 7.74–7.68 (m, 1H, Ar), 7.42–7.35 (m, 2H, Ar), 7.03–6.95 (m, 2H, CHCF), 5.18 (d, 2H, *J* = 4.3 Hz, CH_2_), 4.60 (s, 2H, CH_2_S), 3.73 (s, 3H, CH_3_); ^13^C NMR (CDCl_3_): *δ* = 197.5 (CS), 163.5 (CF), 159.5 (C4 phthalazine), 139.8, 133.6, 132.5, 132.3, 130.9, 130.8, 128.0, 127.7, 127.5 (C5 phthalazine), 123.6, 115.7 (*C*HCF), 115.5 (*C*HCF), 47.5 (CH_2_), 39.5 (CH_3_), 39.4 (CH_2_S); HRMS (ESI): *m/z* [M+H]^+^ calcd for C_18_H_17_FN_3_OS_2_: 374.07916, found: 374.07870.

*4-Chlorobenzyl N-((3-methyl-4-oxo-3,4-dihydrophthalazin-1-yl)methyl)dithiocarbamate* (**9d**). White solid; yield: 68%; R*_f_* = 0.2 (hexane/EtOAc, 2:1); ^1^H NMR (CDCl_3_): *δ* = 8.43 (d, 2H, *J* = 7.7 Hz, H5 phthalazine), 8.23 (br s, 1H, NH), 7.85–7.77 (m, 3H, H6, H7, H8 phthalazine), 7.35 (d, 2H, *J* = 8.4 Hz, Ar), 7.28 (d, 2H, *J* = 8.4 Hz, Ar), 5.19 (d, 2H, *J* = 4.2 Hz, CH_2_), 4.59 (s, 2H, CH_2_S), 3.81 (s, 3H, CH_3_); ^13^C NMR (CDCl_3_): *δ* = 197.2 (CS), 159.4 (C4 phthalazine), 139.7, 135.3, 133.5, 133.4, 132.2, 130.4, 128.7, 127.8, 127.5, 127.3, 123.5, 47.5 (CH_2_), 39.4 (CH_3_), 39.2 (CH_2_S); HRMS (ESI): *m/z* [M+H]^+^ calcd for C_18_H_17_ClN_3_OS_2_: 390.04961, found: 390.04919.

*4-Bromobenzyl N-((3-methyl-4-oxo-3,4-dihydrophthalazin-1-yl)methyl)dithiocarbamate* (**9e**). White solid; yield: 85%; R*_f_* = 0.2 (hexane/EtOAc, 2:1); ^1^H NMR (CDCl_3_): *δ* = 8.48–8.20 (m, 2H, NH, H5 phthalazine), 7.84–7.71 (m, 3H, H6, H7, H8 phthalazine), 7.43 (d, 2H, *J* = 8.2 Hz, Ar), 7.29 (d, 2H, *J* = 8.2 Hz, Ar), 5.18 (d, 2H, *J* = 4.0 Hz, CH_2_), 4.57 (s, 2H, CH_2_S), 3.78 (s, 3H, CH_3_); ^13^C NMR (CDCl_3_): *δ* = 197.2 (CS), 159.5 (C4 phthalazine), 139.8, 136.0, 133.6, 132.3, 131.8, 130.9, 128.0, 127.7, 127.4 (C5 phthalazine), 123.6, 121.6 (CBr), 47.6 (CH_2_), 39.5 (CH_3_), 39.4 (CH_2_S); HRMS (ESI): *m/z* [M+H]^+^ calcd for C_18_H_17_BrN_3_OS_2_: 433.99909, found: 433.99869.

*4-Nitrobenzyl N-((3-methyl-4-oxo-3,4-dihydrophthalazin-1-yl)methyl)dithiocarbamate* (**9f**). White solid; yield: 66%; R*_f_* = 0.2 (hexane/EtOAc, 2:1); ^1^H NMR (DMSO-*d*_6_): *δ* = 10.54 (br s, 1H, NH), 8.32–8.28 (m, 1H, H5 phthalazine), 8.15 (d, 2H, *J* = 8.7 Hz, CHCNO_2_), 7.94–7.85 (m, 3H, H6, H7, H8 phthalazine), 7.63 (d, 2H, *J* = 8.7 Hz, Ar), 5.13 (s, 2H, CH_2_), 4.69 (s, 2H, CH_2_S), 3.71 (s, 3H, CH_3_); ^13^C NMR (DMSO): *δ* = 195.9 (CS), 158.5 (C4 phthalazine), 146.5, 146.0, 140.8, 133.4, 132.0, 130.0, 128.3, 127.0, 126.3 (C5 phthalazine), 124.5, 123.4 (*C*HCNO_2_), 48.1 (CH_2_), 39.0 (CH_3_), 37.3 (CH_2_S); HRMS (ESI): *m/z* [M+H]^+^ calcd for C_18_H_17_N_4_O_3_S_2_: 401.07366, found: 401.07356.

*Prop-2-ynyl N-((3-methyl-4-oxo-3,4-dihydrophthalazin-1-yl)methyl)dithiocarbamate* (**9g**). White solid; yield: 36%; R*_f_* = 0.2 (hexane/EtOAc, 2:1); ^1^H NMR (CDCl_3_): *δ* = 8.48–8.39 (m, 2H, NH, H5 phthalazine), 7.86–7.76 (m, 3H, H6, H7, H8 phthalazine), 5.19 (d, 2H, *J* = 4.2 Hz, CH_2_), 4.10 (d, 2H, *J* = 2.6 Hz, CH_2_S), 3.81 (s, 3H, CH_3_), 2.6 (t, 1H, *J* = 2.6 Hz, HC≡); ^13^C NMR (CDCl_3_): *δ* = 195.7 (CS), 159.5 (C4 phthalazine), 139.4, 133.6, 132.4, 127.5 (C5 phthalazine), 123.5, 78.4 (C≡), 72.2 (HC≡), 47.5 (CH_2_), 39.5 (CH_3_), 24.3 (CH_2_S); HRMS (ESI): *m/z* [M+H]^+^ calcd for C_14_H_14_N_3_OS_2_: 304.05728, found: 304.05719.

### 3.3. Antiproliferative Activity Studies

Antiproliferative effects of titled compounds on NCI-H460, A2780, and MCF-7 cells were studied using the colorimetric MTT assay [40,41,42]. All the cell lines were acquired from ATCC. Cells (passages between 15 and 18) were seeded in 96-well plates (15,000 cells per well for the NCI-H460 cell line, 4000 for the A2780 cell line, and 10,000 for the MCF-7 cell line), incubated for 24 h in the culture medium (RPMI1640 supplemented with 10% FBS and 2 mM L-glutamine for both NCI-H460 and A2780 cell lines and EMEM supplemented with 10% FBS and 0.01 mg/mL of bovine insulin for MCF-7 cell line), and treated at 37 °C for 96 h (A2780 and MCF-7) and 48 h (NCI-H460) with concentrations in the range of 0.1–100 µM of target compounds and the reference drug (cisplatin) dissolved in DMSO. Three wells were used for each of the variants tested. After the incubation time, aliquots of MTT solution in phosphate-buffered saline (10 μL) were added to each well and incubated for 4 h. The color formed was quantified by a spectrophotometric plate reader (Tecan Ultra evolution) at 595 nm wavelength. In all experiments, DMSO controls were included. The percentage of inhibition of cell viability was calculated by the formula % inhibition = 100 − ((AO × 100)/AT), where AO is the absorbance observed in the treated wells and AT is the absorbance observed in the DMSO control wells.

The antiproliferative potency of the studied compounds was determined from concentration–effect curves, by using GraphPad Prism software (version 2.01), and was expressed as 50% inhibitory concentrations (IC_50_). Correlation coefficients (r^2^) were higher than 0.995 for all the compounds tested.

### 3.4. Drug-Like and Toxicity Properties Prediction

Physicochemical properties, drug-likeness, and ADME of new phthalazinone-dithiocarbamate hybrids were predicted by using the free web-server SwissADME [43]. The 2D structure of compounds was drawn using ChemDraw Professional 18.2.0.48 to obtain the corresponding SMILE code, which was inserted into the SwissADME website. The software computes many parameters affecting pharmacokinetics, interaction with carriers and CYP450 enzymes, and drug-like properties. In addition, it provides two kinds of diagrams, the bioavailability radar for a quick drug-likeness appraisal, and the BOILED-Egg tool for graphical estimation of HIA and BBB permeation.

Acute toxicity, organ toxicity (hepatotoxicity), and toxicity endpoints (carcinogenicity, immunotoxicity, mutagenicity, and cytotoxicity) were computed using the webserver ProTox-II [44]. This free virtual lab incorporates molecular similarity for DL_50_ prediction and pharmacophore-based models as well as fragment propensities and machine learning models for the prediction of hepatotoxicity and several toxicity endpoints. The website takes the 2D structure to estimate the toxicity of small molecules.

## 4. Conclusions

Two novel series of phthalazinone-dithiocarbamate hybrids were successfully obtained and characterized, compounds **6**–**8** containing the dithiocarbamate group at N2 and compounds **9** displaying this fragment at C4. The antiproliferative activity of all synthesized compounds was evaluated against three human cancer cell lines (A-2780, NCI-H460, MCF-7). Among compounds **6**–**8**, the 4-bromobenzyl derivatives **6e**, **8e**, and the propargyl analogue **6g** had good activity versus the A-2780 cell line, with IC_50_ values of 5.53 ± 0.09 µM, 7.51 ± 0.13 µM, and 5.20 ± 0.13 µM, respectively. Compound **6g** also showed good antiproliferative potential against the MCF-7 cell line (IC_50_ = 7.64 ± 0.5 µM).

On the other hand, most of compounds **9** had good activity and selectivity versus the NCI-H460 cell line, with IC_50_ values below or around 10 µM, the benzyl analogues **9a**–**b** and **9d** were the most promising. Interestingly, the propargyl group in compounds **9** also provided the best result against the A-2780 cell line, compound **9g** with an IC_50_ value of 6.75 ± 0.12 µM was the most active derivative against this ovarian cancer cell line A-2780.

Drug-like and toxicity predictions suggested interesting properties for the studied phthalazinone-dithiocarbamate hybrids, specifically regarding oral absorption, bioavailability, absence of effects on the CNS, and the toxicological profile. All of this seems to indicate that compounds **6e**, **8e**, **6g**, **9a**–**b**, **9d**, and **9g** could have good potential for the future development of anticancer agents.

## Figures and Tables

**Table 1:** Antiproliferative activity (IC_50_ values) of phthalazinone-dithiocarbamate hybrid compounds **6a**–**g**, **7a**–**g**, and **8a**–**g** against A-2780, NCI-H460, and MCF-7 cell lines.

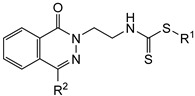				IC_50_^a^ (µM)	
Compound	R^2^	R^1^	A-2780	NCI-H460	MCF-7
**6a**	H	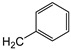	88 ± 15	>100	75 ± 1
**7a**	*p*-Tol	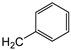 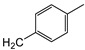	10 ± 1	>100	33 ± 1
**8a**	Me		29 ± 3	>100	>100
**6b**	H		78 ± 6	>100	22 ± 2
**7b**	*p*-Tol	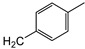 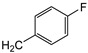	17 ± 1	>100	26 ± 1
**8b**	Me		27 ± 1	>100	37 ± 3
**6c**	H		61 ± 6	>100	36 ± 6
**7c**	*p*-Tol	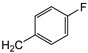 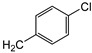	15 ± 1	>100	51 ± 1
**8c**	Me		44 ± 7	>100	>100
**6d**	H		15 ± 1	>100	46 ± 1
**7d**	*p*-Tol	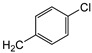 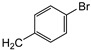	20 ± 1	>100	>100
**8d**	Me		19 ± 1	>100	24 ± 1
**6e**	H		5.53 ± 0.09	>100	20 ± 1
**7e**	*p*-Tol	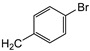 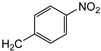	17 ± 1	>100	>100
**8e**	Me		7.51 ± 0.13	>100	>100
**6f**	H		55 ± 10	>100	25 ± 1
**7f**	*p*-Tol	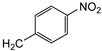 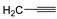	10 ± 1	>100	40 ± 1
**8f**	Me		11 ± 1	>100	14 ± 1
**6g**	H		5.20 ± 0.13	>100	7.64 ± 0.5
**7g**	*p*-Tol	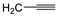 -	24 ± 1	43 ± 1	28 ± 1
**8g**	Me		23 ± 1	>100	ND
**Cisplatin**	-		0.54 ± 0.01	5.54 ± 0.23	13 ± 1

^a^ Data are expressed as the mean ± standard error from three independent experiments (*n* = 3).

**Table 2:** Antiproliferative activity (IC_50_ values) of phthalazinone-dithiocarbamate hybrid compounds **9a**–**g** against A-2780, NCI-H460, and MCF-7 cell lines.

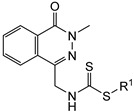			IC_50_^a^ (µM)	
Compound	R^1^	A-2780	NCI-H460	MCF-7
**9a**	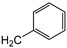	12 ± 1	7.36 ± 0.08	12 ± 1
**9b**	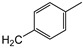	32 ± 3	8.49 ± 0.25	10 ± 1
**9c**	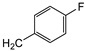	43 ± 4	12 ± 1	84 ± 15
**9d**	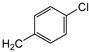	34 ± 5	7.77 ± 0.17	39 ± 4
**9e**	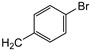	24 ± 1	12 ± 1	33 ± 2
**9f**	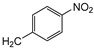	12 ± 1	25 ± 1	62 ± 2
**9g**	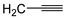	6.75 ± 0.12	34 ± 1	29 ± 2
**Cisplatin**	-	0.54 ± 0.01	5.54 ± 0.23	13 ± 1

^a^ Data are expressed as the mean ± standard error from three independent experiments (*n* = 3).

**Table 3:** Physicochemical and lipophilicity properties.

Compound	Molecular Weight (g/mol)	Heavy Atoms	Arom. Heavy Atoms	Fraction Csp^3^	Rotable Bonds	H-Bond Aceptors	H-Bond Donors	Molar Refractivity	TPSA (Å^2^)	Log P_o/w_
**6e**	434.37	25	16	0.17	7	2	1	112.06	104.31	3.92
**8e**	448.40	26	16	0.21	7	2	1	117.02	104.31	4.24
**6g**	303.40	20	10	0.21	6	2	1	87.65	104.31	2.28
**9a**	355.48	24	16	0.17	6	2	1	104.52	104.31	3.36
**9b**	369.50	25	16	0.21	6	2	1	109.48	104.31	3.73
**9d**	389.92	25	16	0.17	6	2	1	109.53	104.31	3.85
**9g**	288.39	19	10	0.31	4	2	0	85.00	92.28	2.94

**Table 4:** Acute toxicity results obtained from ProTox-II web platform.

Compound	DL_50_ (mg/kg)	Toxicity Class	Average Similarity (%)	Prediction Accuracy (%)
**6e**	350	4	47.19	54.26
**8e**	350	4	53.95	67.38
**6g**	350	4	46.36	54.26
**9a**	500	4	47.02	54.26
**9b**	500	4	47.63	54.26
**9d**	500	4	49.72	54.26
**9g**	500	4	47.18	54.26

## Data Availability

Data are contained within the article or Supplementary Material.

## Supplementary Materials

The following supporting information can be downloaded at: https://www.mdpi.com/article/10.3390/molecules27238115/s1, Table S1: CYP450 inhibition profile of compounds **6e**, **8e**, **6g**, **9a**–**b**, **9d**, and **9g**; Table S2: Toxicity model predicted by ProTox-II for compounds **6e**, **8e**, **6g**, **9a**–**b**, **9d**, and **9g**; synthesis of compounds **15**–**17**; copies of ^1^H NMR and ^13^C NMR of compounds **6**–**9**.

## Sample Availability

Samples of the compounds are available from the authors.
