# Supplementary material for: Novel Phthalazin-1(2H)-One Derivatives Displaying a Dithiocarbamate Moiety as Potential Anticancer Agents
Source: Molecules. 2022 Nov 22;27(23):8115. doi: 10.3390/molecules27238115 (PMC9738785; doi:10.3390/molecules27238115)
Supplement: Supplementary file 1 [file molecules-27-08115-s001.zip › molecules-2034416-supplementary.pdf]

Supplementary Materials

Novel phthalazin-1(2H)-one derivatives displaying a dithiocarbamate moiety as potential anticancer agents

Table of contents

Results and discussion

Table S1. CYP450 inhibition profile of compounds **6e**, **8e**, **6g**, **9a-b**, **9d** and **9g** 1

Table S2. Toxicity model profile of compounds **6e**, **8e**, **6g**, **9a-b**, **9d** and **9g** 2

Materials and Methods

Chemical synthesis

Synthesis of 2-(2-bromoethyl)phthalazinones **15-17** 2

^{1}H NMR and ^{13}C NMR spectra of compounds **6-9** 3

Table S1. CYP450 inhibition predicted by SwissADME for compounds **6e**, **8e**, **6g**, **9a-b**, **9d**, and **9g**.

Compound	CYP1A2	CYP2C19	CYP2C9	CYP2D6	CYP3A4
**6e**	Yes	Yes	Yes	No	Yes
**8e**	Yes	Yes	Yes	No	Yes
**6g**	Yes	Yes	Yes	No	No
**9a**	Yes	Yes	Yes	No	Yes
**9b**	Yes	Yes	Yes	No	Yes
**9d**	Yes	Yes	Yes	No	Yes
**9g**	Yes	Yes	Yes	No	No

Table S2. Toxicity model predicted by ProTox-II for compounds **6e**, **8e**, **6g**, **9a-b**, **9d,** and **9g**.

Compound	Hepatotoxicity Probability	Carcinogenicity Probability	Immunotoxicity Probability	Mutagenicity Probability	Cytotoxicity Probability
**6e**	Inactive	Inactive	Inactive	Inactive	Inactive
	0.56	0.55	0.80	0.53	0.67
**8e**	Inactive	Inactive	Inactive	Inactive	Inactive
	0.58	0.56	0.98	0.51	0.67
**6g**	Inactive	Active	Inactive	Inactive	Inactive
	0.54	0.53	0.84	0.53	0.72
**9a**	Inactive	Active	Inactive	Inactive	Inactive
	0.57	0.53	0.98	0.51	0.71
**9b**	Inactive	Active	Inactive	Inactive	Inactive
	0.58	0.53	0.99	0.51	0.71
**9d**	Inactive	Inactive	Inactive	Inactive	Inactive
	0.58	0.57	0.93	0.52	0.6
**9g**	Inactive	Active	Inactive	Inactive	Inactive
	0.55	0.53	0.92	0.51	0.72

Materials and Methods

Chemical synthesis

General procedure for the preparation of 2-(2-bromoethyl)phthalazin-1(2*H*)-ones (15-17)

To a solution of phthalazinone **12-14** (2 mmol) in DMF (20 mL) was added K_2CO_3 (8 mmol) and 1,2-dibromoethane (12 mmol). The reaction mixture was stirred at 60 ºC for 1.5 h and the solvent was evaporated to dryness. The residue was purified by column chromatography on silica gel (hexane/EtOAc, 5:1) to afford the desired compound.

2-(2-Bromoethyl)phthalazin-1(2H)-one (**15**). White solid; yield: 50%; $R_f = 0.4$ (hexane/EtOAc, 1:1); ^{1}H NMR ($CDCl_3$): $\delta = 8.43$ (d, 1H, $J = 7.7$ Hz, H8), 8.18 (s, 1H, H4), 7.85-7.76 (m, 2H, H6, H7), 7.71 (d, 1H, $J = 7.3$ Hz, H5), 4.63 (t, 2H, $J = 6.8$ Hz, H1´), 3.77 (t, 2H, $J = 6.8$ Hz, H2´); ^{13}C NMR ($CDCl_3$): $\delta = 159.6$ (C1), 138.3, 133.5, 132.0, 129.8, 127.9, 126.9 (C8), 126.3, 52.2 (C1´), 28.6 (C2´); HRMS (ESI): *m/z* $[M+H]^+$ calcd for $C_{10}H_{10}BrN_2O$: 252.99710, found: 252.99790.

2-(2-Bromoethyl)-4-p-tolylphthalazin-1(2H)-one (**16**). White solid; yield: 75%; $R_f = 0.4$ (hexane/EtOAc, 1:1); ^{1}H NMR ($CDCl_3$): $\delta = 8.53$ (d, 1H, $J = 7.1$ Hz, H8), 7.82-7.75 (m, 3H, H5,

H6, H7), 7.49 (d, 2H, *J* = 7.9 Hz, Ar), 7.34 (d, 2H, *J* = 7.9 Hz, Ar), 4.69 (t, 2H, *J* = 7.0 Hz, H1′), 3.80 (t, 2H, *J* = 7.0 Hz, H2′), 2.46 (s, 3H, CH_3); ^{13}C NMR ($CDCl_3$): δ = 159.3 (C1), 147.7, 139.5, 133.1, 132.1, 131.6, 129.5, 129.4, 129.3, 128.2, 127.4, 127.0, 52.1 (C1′), 28.5 (C2′), 21.5 (CH_3); HRMS (ESI): *m/z* $[M+H]^+$ calcd for $C_{17}H_{16}BrN_2O$: 343.04405, found: 343.04401.

2-(2-Bromoethyl)-4-methylphthalazin-1(2H)-one (**17**). White solid; yield: 45%; R_f = 0.4 (hexane/EtOAc, 1:1); ^1H NMR ($CDCl_3$): δ = 8.45-8.41 (m, 1H, H8), 7.83-7.72 (m, 3H, H5, H6, H7), 4.56 (t, 2H, *J* = 7.0 Hz, H1′), 3.73 (t, 2H, *J* = 7.0 Hz, H2′), 2.57 (s, 3H, CH_3); ^{13}C NMR ($CDCl_3$): δ = 159.4 (C1), 144.1 (C4), 133.2, 131.5, 129.9, 127.7, 127.2, 125.0, 51.8 (C1′), 28.5 (C2′), 19.0 (CH_3); HRMS (ESI): *m/z* $[M+H]^+$ calcd for $C_{11}H_{12}BrN_2O$: 267.01275, found: 267.01300.

^1H NMR and ^{13}C NMR spectra of compounds 6-9

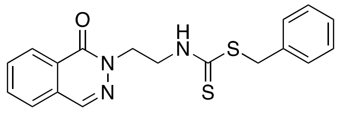

6a

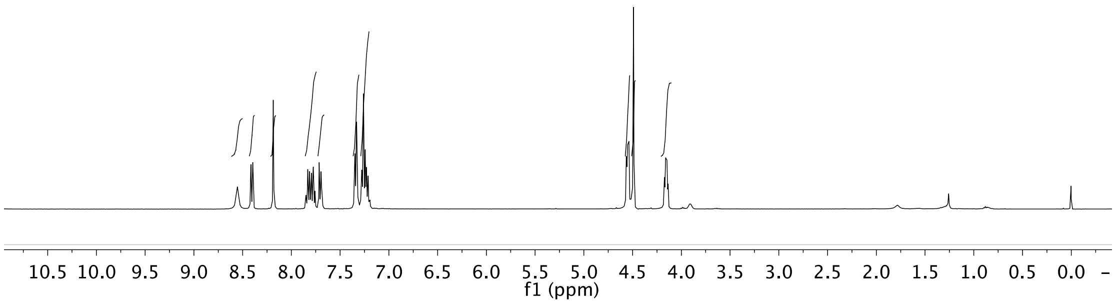

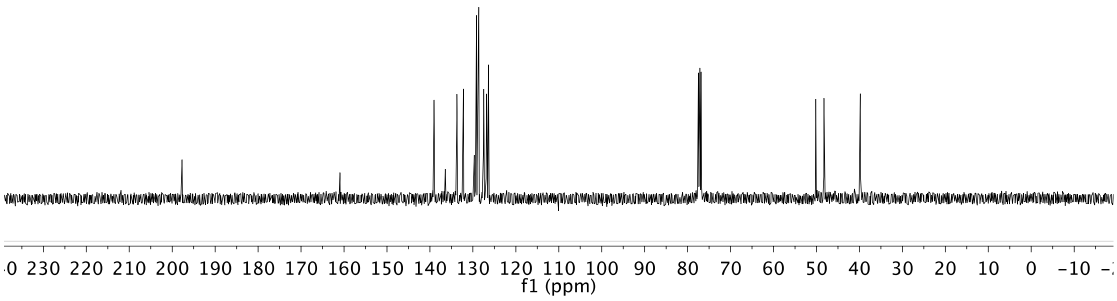

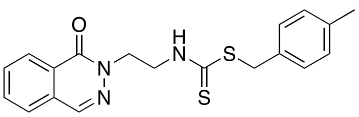

6b

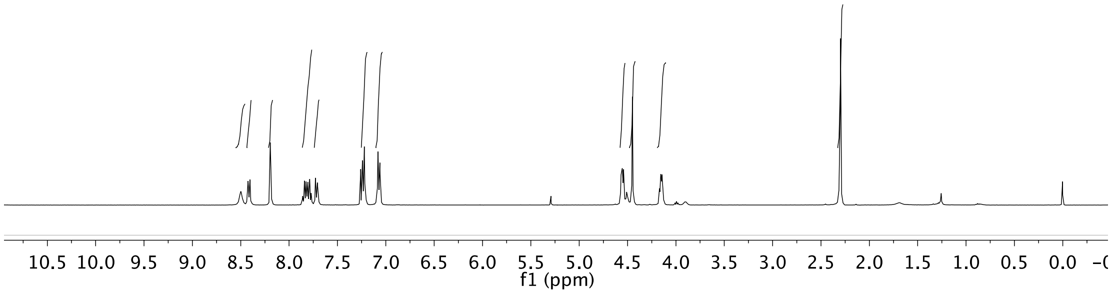
10.5 10.0 9.5 9.0 8.5 8.0 7.5 7.0 6.5 6.0 5.5 5.0 4.5 4.0 3.5 3.0 2.5 2.0 1.5 1.0 0.5 0.0
f1 (ppm)

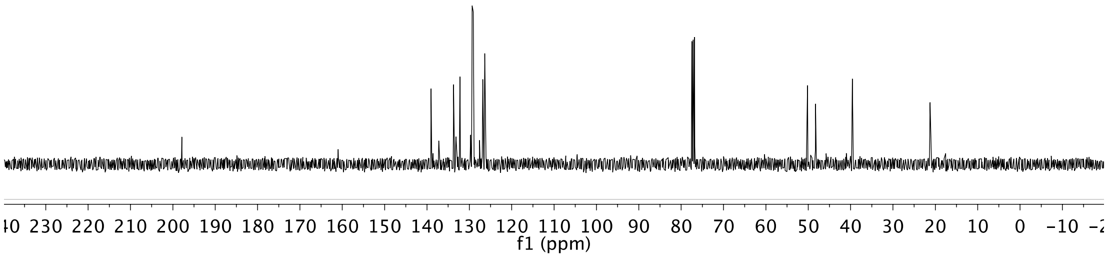
230 220 210 200 190 180 170 160 150 140 130 120 110 100 90 80 70 60 50 40 30 20 10 0 −10
f1 (ppm)

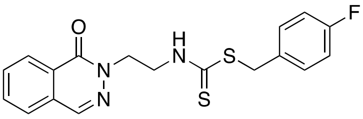

6c

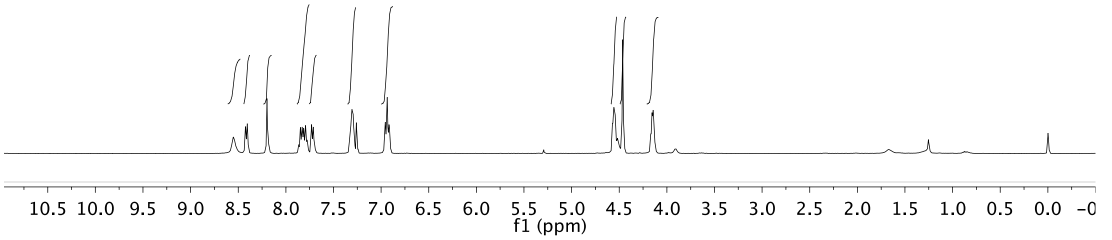
10.5 10.0 9.5 9.0 8.5 8.0 7.5 7.0 6.5 6.0 5.5 5.0 4.5 4.0 3.5 3.0 2.5 2.0 1.5 1.0 0.5 0.0
f1 (ppm)

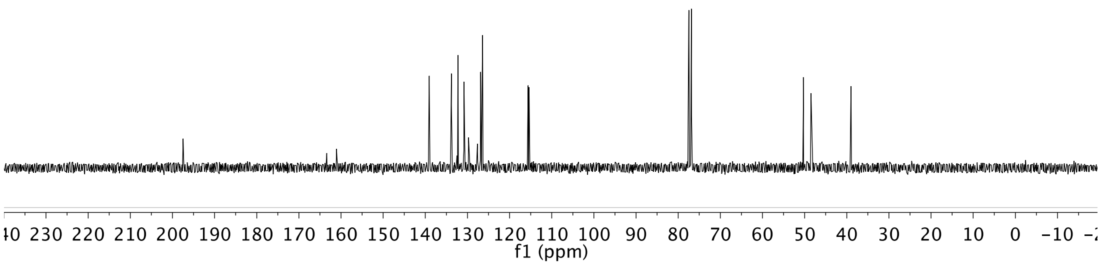
230 220 210 200 190 180 170 160 150 140 130 120 110 100 90 80 70 60 50 40 30 20 10 0 −10
f1 (ppm)

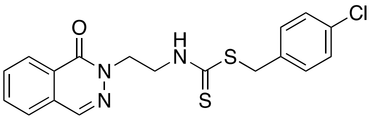

6d

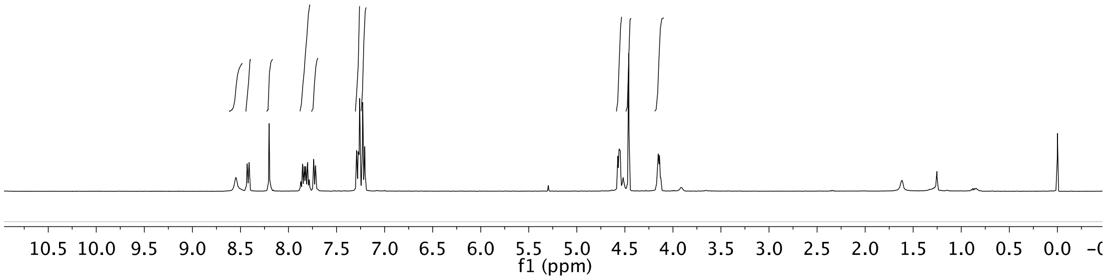
10.5 10.0 9.5 9.0 8.5 8.0 7.5 7.0 6.5 6.0 5.5 5.0 4.5 4.0 3.5 3.0 2.5 2.0 1.5 1.0 0.5 0.0
f1 (ppm)

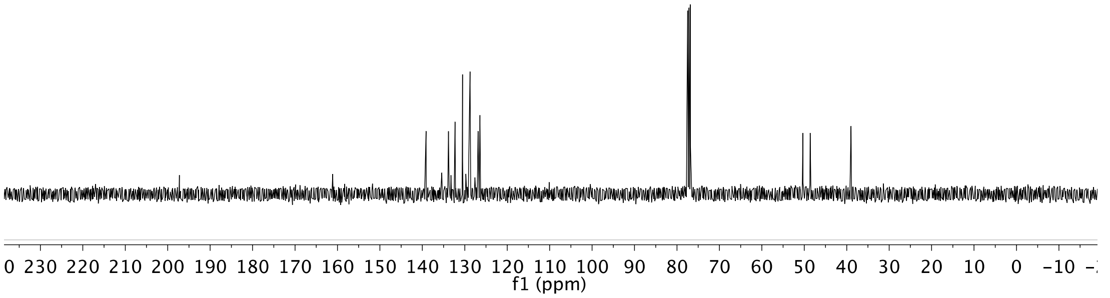
230 220 210 200 190 180 170 160 150 140 130 120 110 100 90 80 70 60 50 40 30 20 10 0 −10
f1 (ppm)

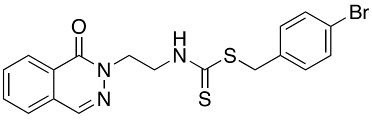

6e

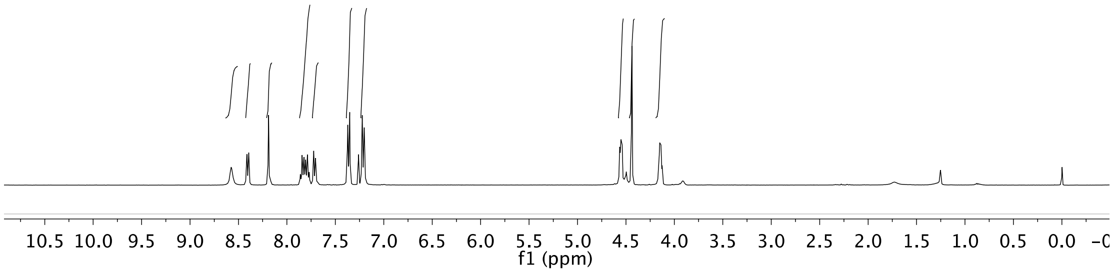
10.5 10.0 9.5 9.0 8.5 8.0 7.5 7.0 6.5 6.0 5.5 5.0 4.5 4.0 3.5 3.0 2.5 2.0 1.5 1.0 0.5 0.0
f1 (ppm)

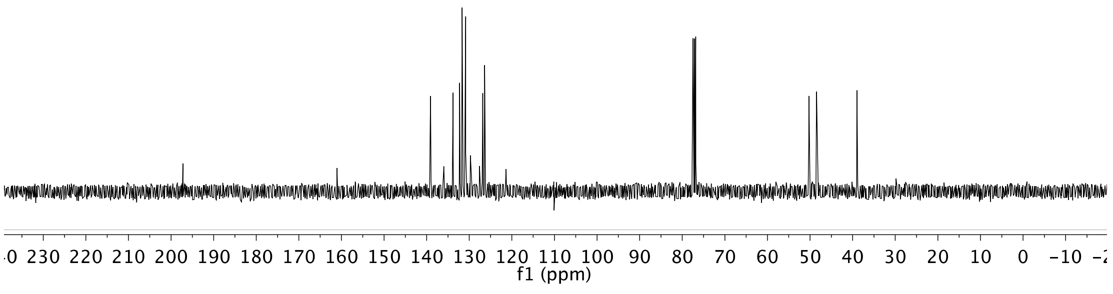
230 220 210 200 190 180 170 160 150 140 130 120 110 100 90 80 70 60 50 40 30 20 10 0 −10
f1 (ppm)

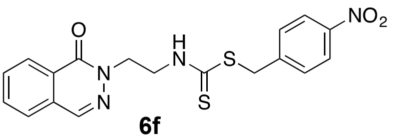
NO2

6f

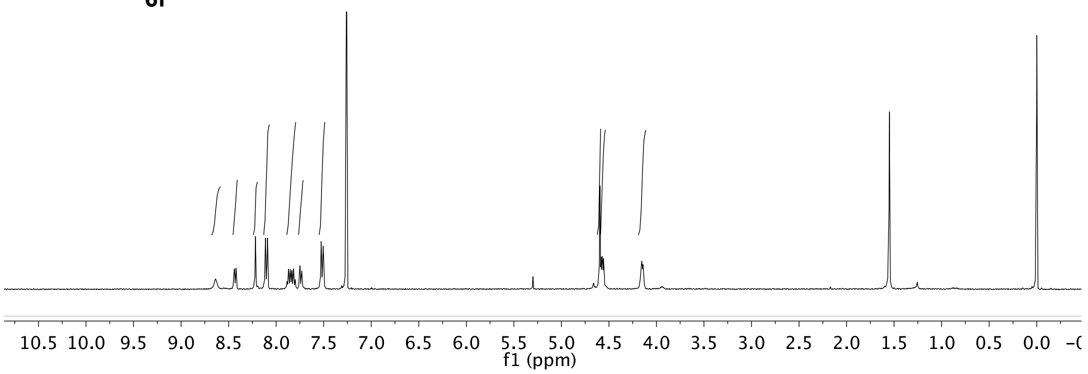
10.5 10.0 9.5 9.0 8.5 8.0 7.5 7.0 6.5 6.0 5.5 5.0 4.5 4.0 3.5 3.0 2.5 2.0 1.5 1.0 0.5 0.0
f1 (ppm)

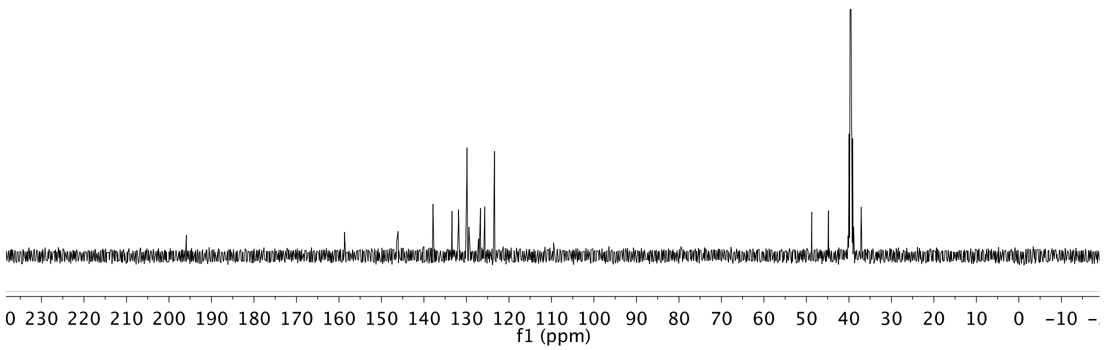
230 220 210 200 190 180 170 160 150 140 130 120 110 100 90 80 70 60 50 40 30 20 10 0 −10
f1 (ppm)

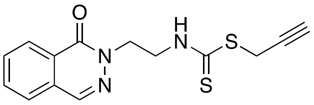

6g

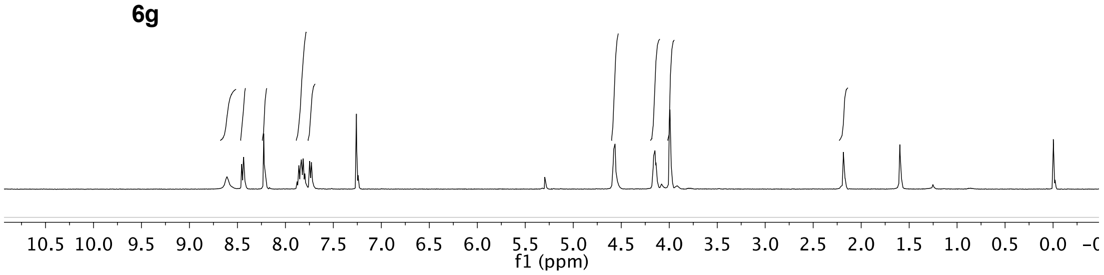
10.5 10.0 9.5 9.0 8.5 8.0 7.5 7.0 6.5 6.0 5.5 5.0 4.5 4.0 3.5 3.0 2.5 2.0 1.5 1.0 0.5 0.0
f1 (ppm)

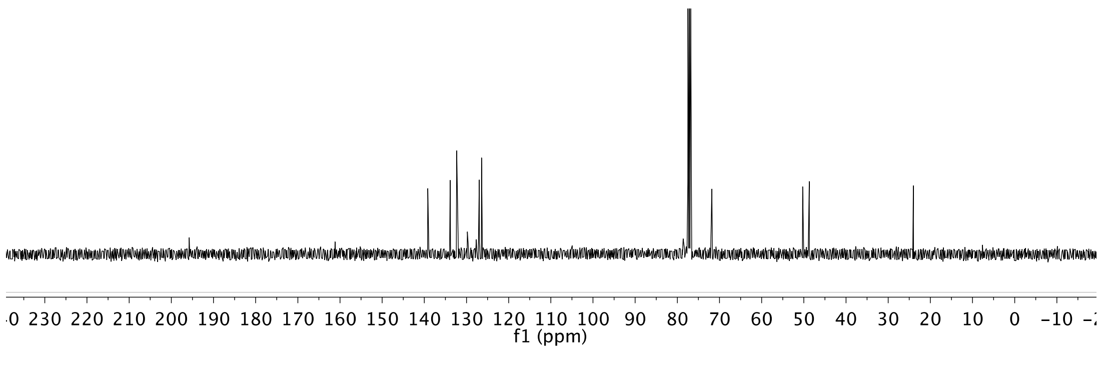
230 220 210 200 190 180 170 160 150 140 130 120 110 100 90 80 70 60 50 40 30 20 10 0 −10
f1 (ppm)

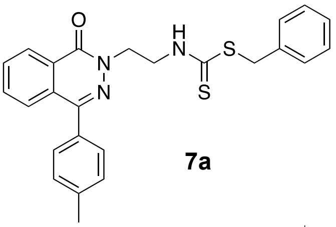

7a

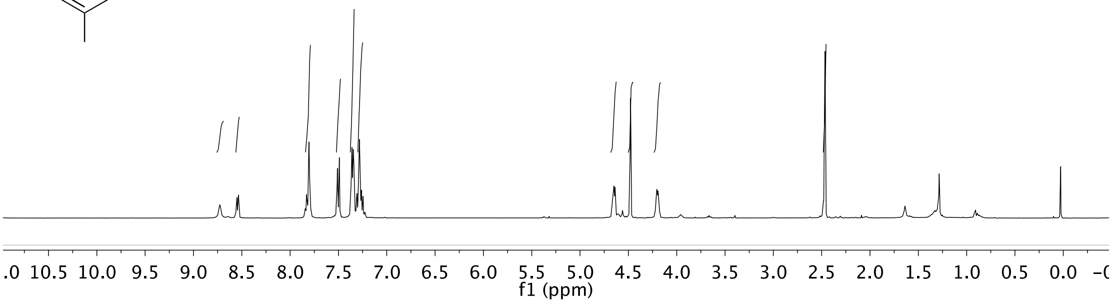

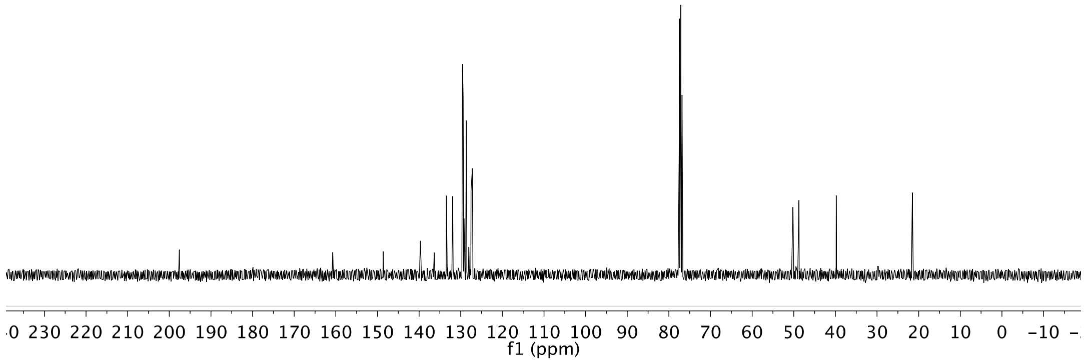

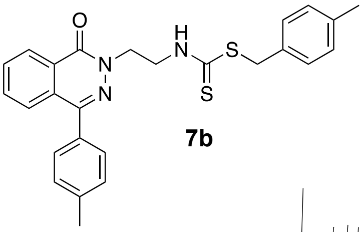

7b

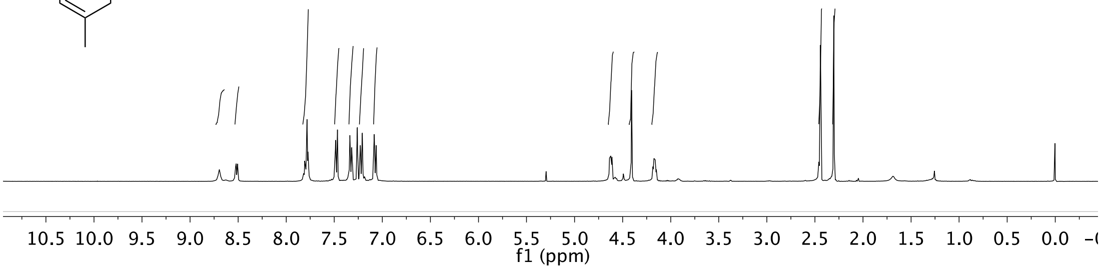

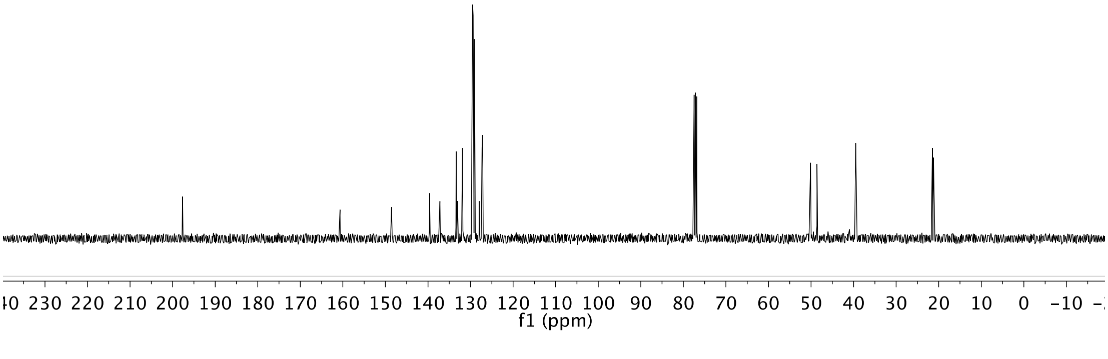

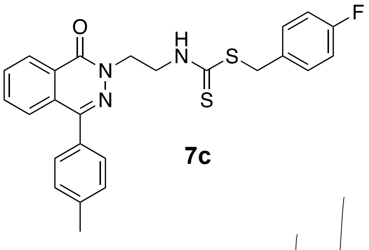
O
N
H
N
S
S
F
N

7c

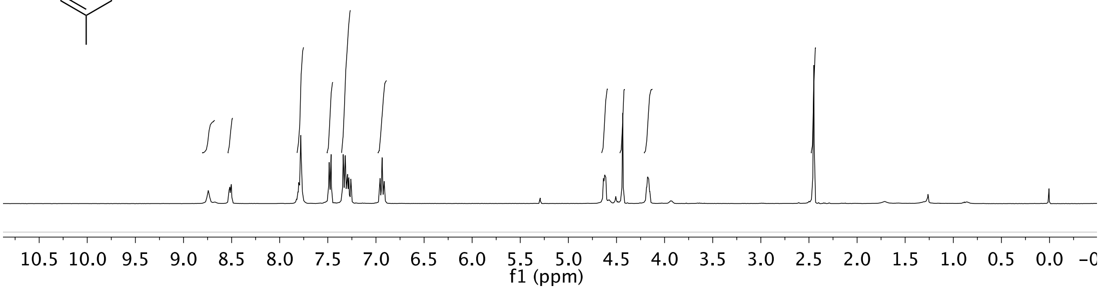
10.5 10.0 9.5 9.0 8.5 8.0 7.5 7.0 6.5 6.0 5.5 5.0 4.5 4.0 3.5 3.0 2.5 2.0 1.5 1.0 0.5 0.0
f1 (ppm)

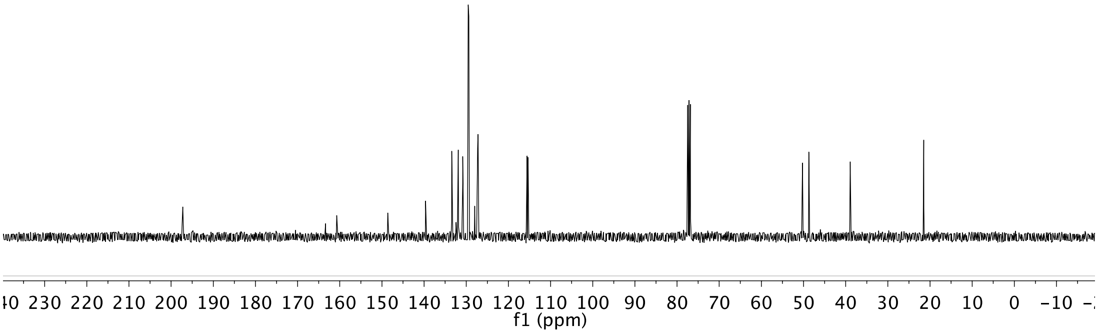
230 220 210 200 190 180 170 160 150 140 130 120 110 100 90 80 70 60 50 40 30 20 10 0 −10
f1 (ppm)

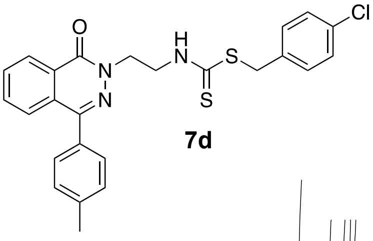
O
N
H
N
S
S
Cl
N

7d

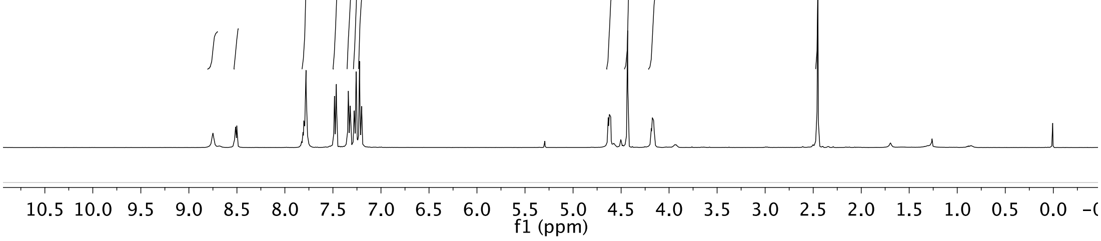
10.5 10.0 9.5 9.0 8.5 8.0 7.5 7.0 6.5 6.0 5.5 5.0 4.5 4.0 3.5 3.0 2.5 2.0 1.5 1.0 0.5 0.0
f1 (ppm)

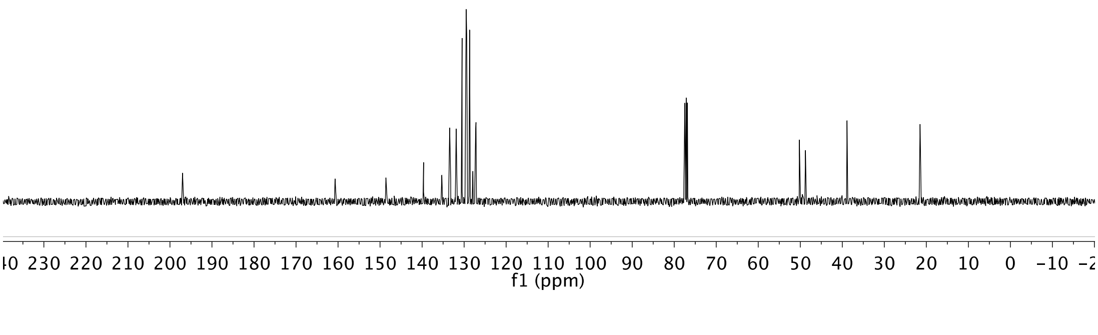
230 220 210 200 190 180 170 160 150 140 130 120 110 100 90 80 70 60 50 40 30 20 10 0 −10
f1 (ppm)

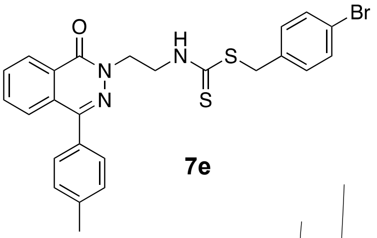
Br
O
H
N
S
N
N
S

7e

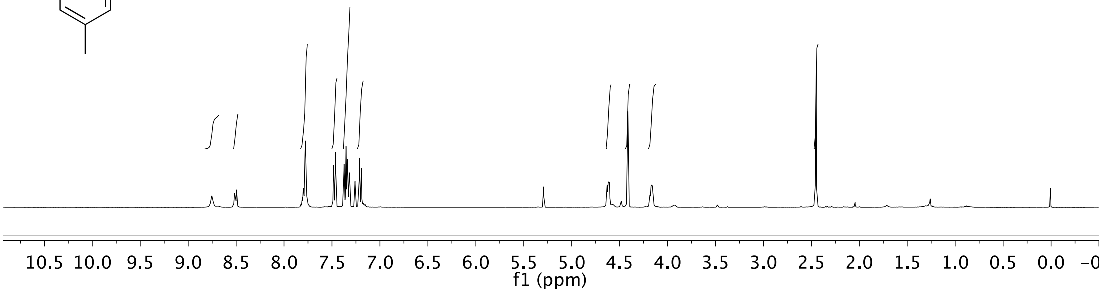
10.5 10.0 9.5 9.0 8.5 8.0 7.5 7.0 6.5 6.0 5.5 5.0 4.5 4.0 3.5 3.0 2.5 2.0 1.5 1.0 0.5 0.0
f1 (ppm)

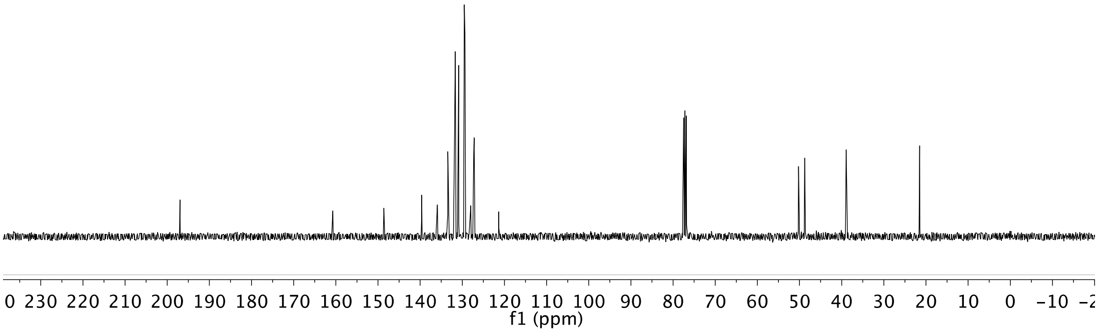
230 220 210 200 190 180 170 160 150 140 130 120 110 100 90 80 70 60 50 40 30 20 10 0 −10
f1 (ppm)

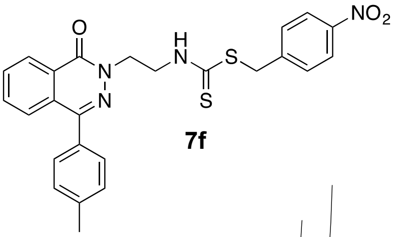
NO_2
O
H
N
S
N
N
S

7f

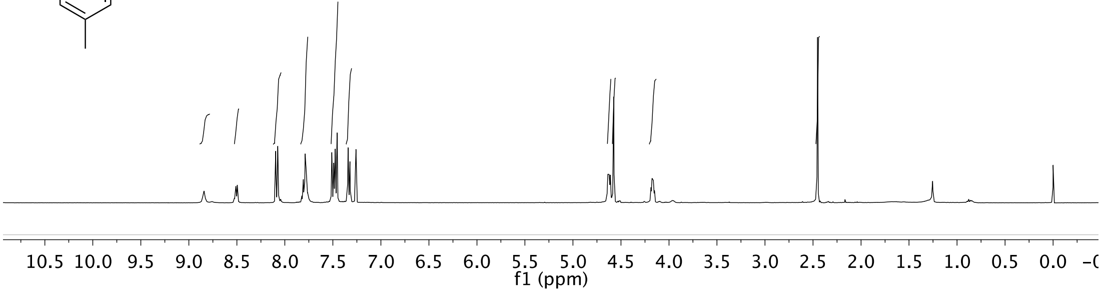
10.5 10.0 9.5 9.0 8.5 8.0 7.5 7.0 6.5 6.0 5.5 5.0 4.5 4.0 3.5 3.0 2.5 2.0 1.5 1.0 0.5 0.0
f1 (ppm)

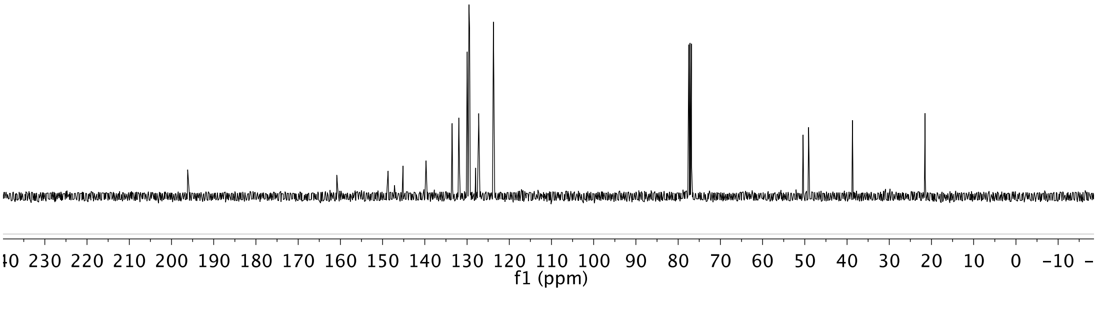
230 220 210 200 190 180 170 160 150 140 130 120 110 100 90 80 70 60 50 40 30 20 10 0 −10
f1 (ppm)

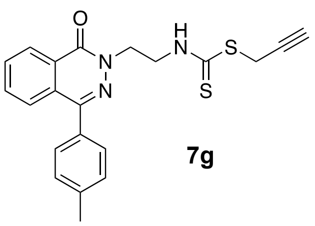

7g

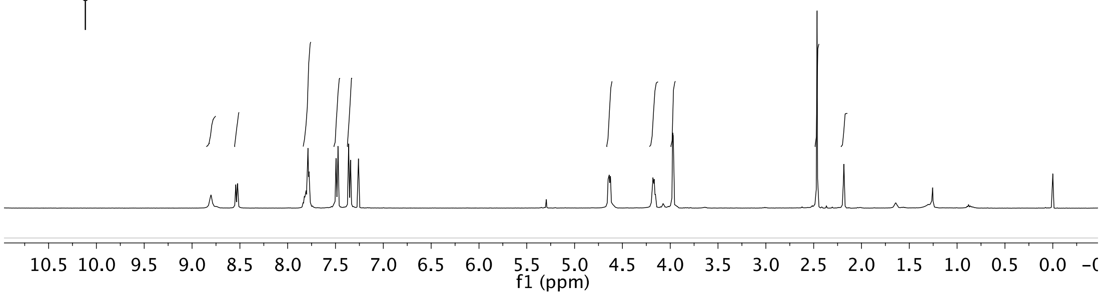
10.5 10.0 9.5 9.0 8.5 8.0 7.5 7.0 6.5 6.0 5.5 5.0 4.5 4.0 3.5 3.0 2.5 2.0 1.5 1.0 0.5 0.0
f1 (ppm)

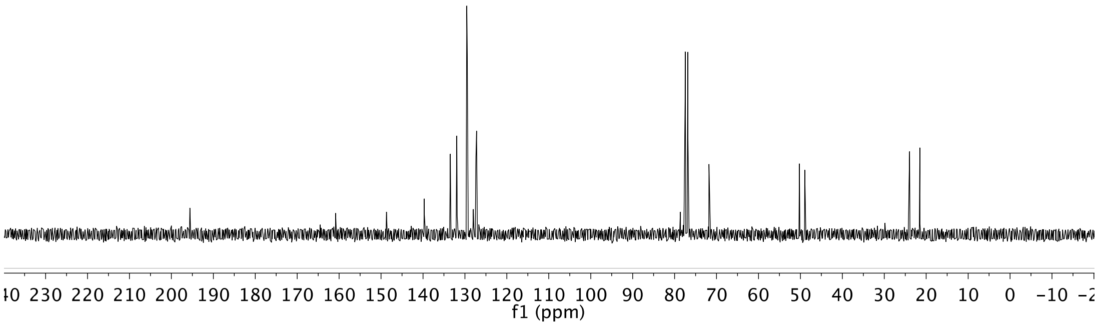
230 220 210 200 190 180 170 160 150 140 130 120 110 100 90 80 70 60 50 40 30 20 10 0 −10
f1 (ppm)

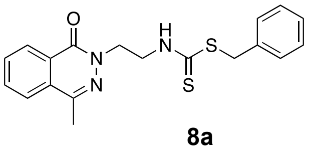

8a

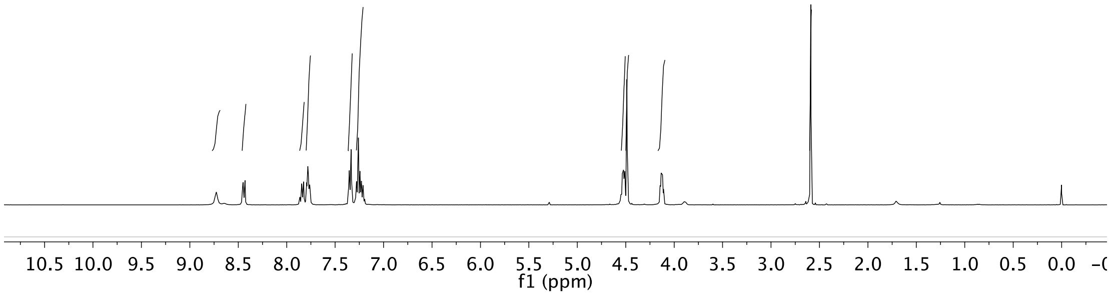
10.5 10.0 9.5 9.0 8.5 8.0 7.5 7.0 6.5 6.0 5.5 5.0 4.5 4.0 3.5 3.0 2.5 2.0 1.5 1.0 0.5 0.0
f1 (ppm)

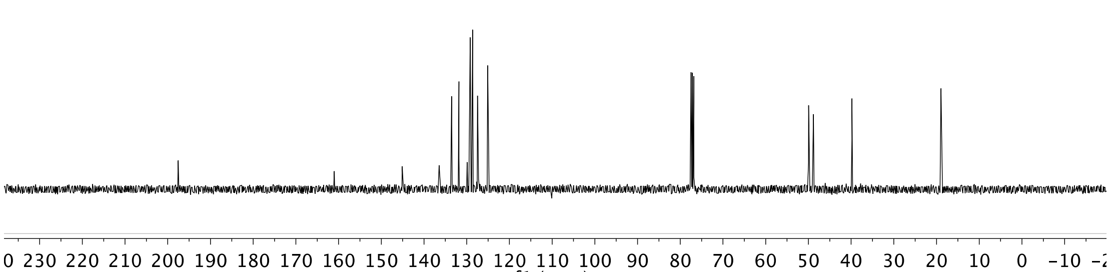
230 220 210 200 190 180 170 160 150 140 130 120 110 100 90 80 70 60 50 40 30 20 10 0 −10
f1 (ppm)

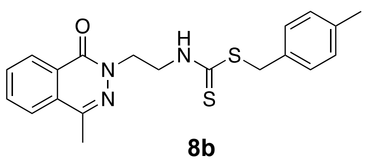

8b

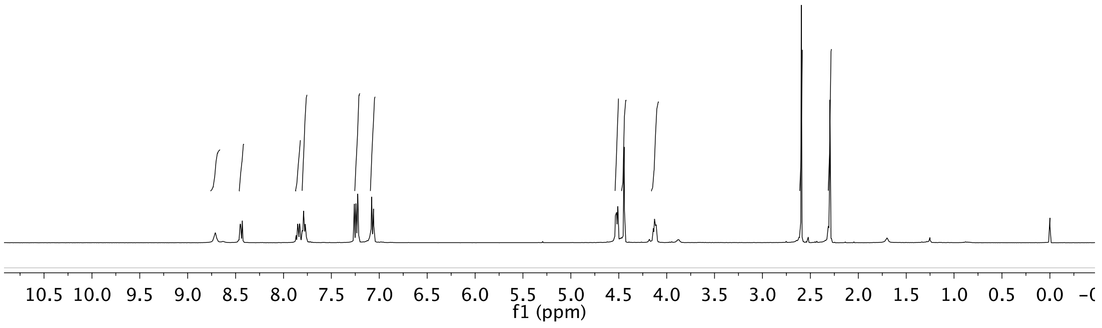
10.5 10.0 9.5 9.0 8.5 8.0 7.5 7.0 6.5 6.0 5.5 5.0 4.5 4.0 3.5 3.0 2.5 2.0 1.5 1.0 0.5 0.0
f1 (ppm)

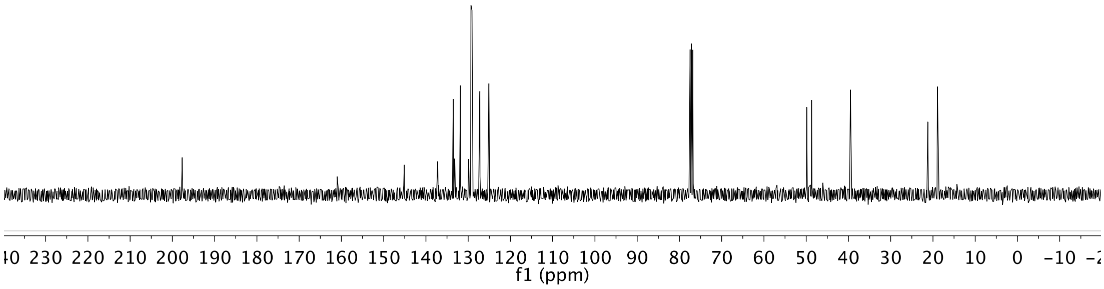
230 220 210 200 190 180 170 160 150 140 130 120 110 100 90 80 70 60 50 40 30 20 10 0 −10
f1 (ppm)

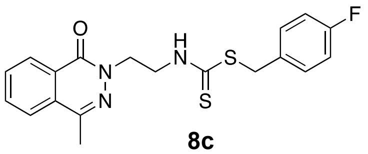

8c

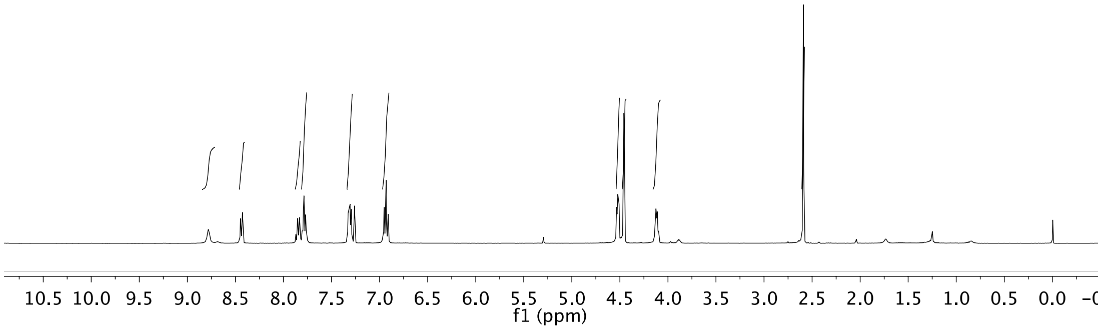
10.5 10.0 9.5 9.0 8.5 8.0 7.5 7.0 6.5 6.0 5.5 5.0 4.5 4.0 3.5 3.0 2.5 2.0 1.5 1.0 0.5 0.0
f1 (ppm)

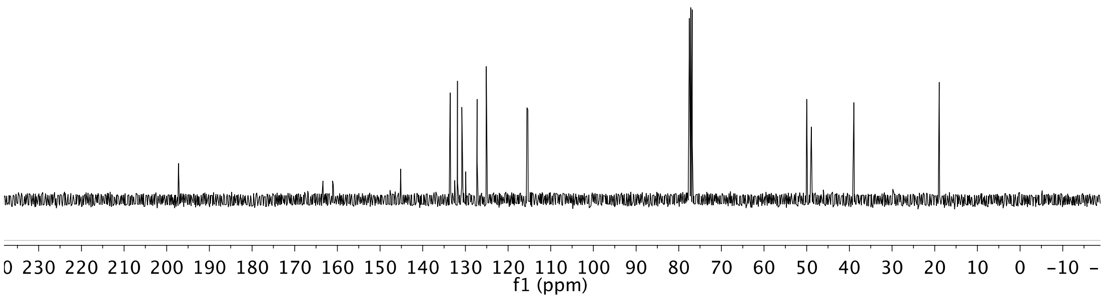
230 220 210 200 190 180 170 160 150 140 130 120 110 100 90 80 70 60 50 40 30 20 10 0 −10
f1 (ppm)

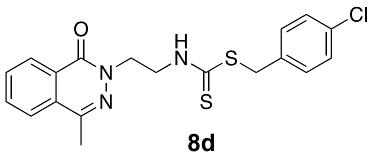

8d

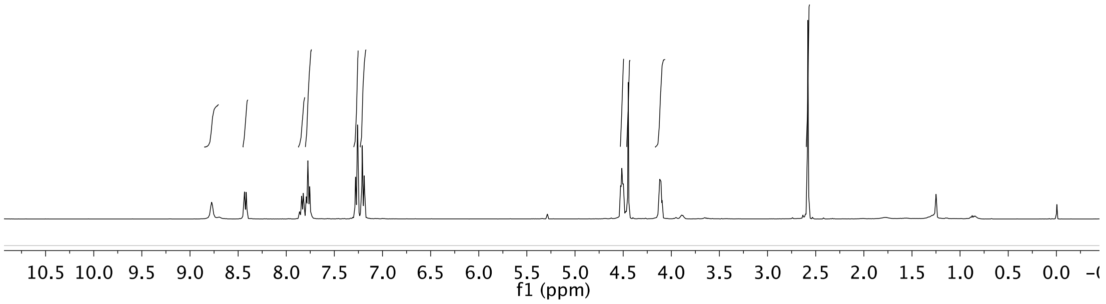

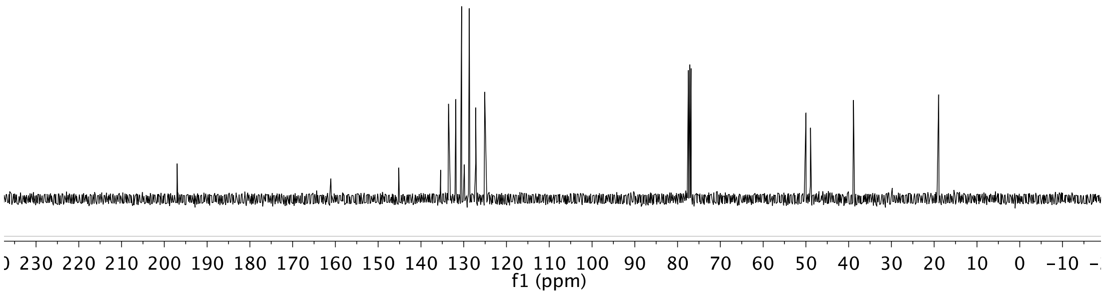

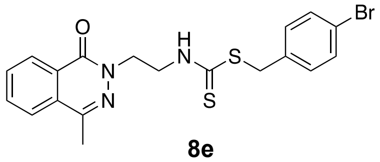

8e

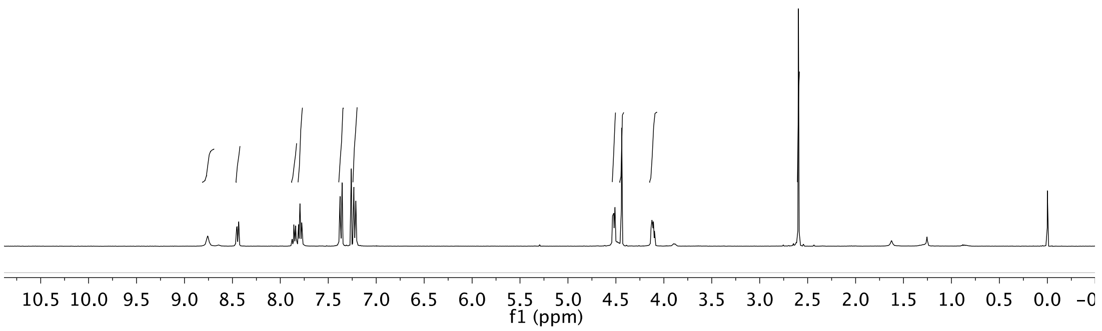

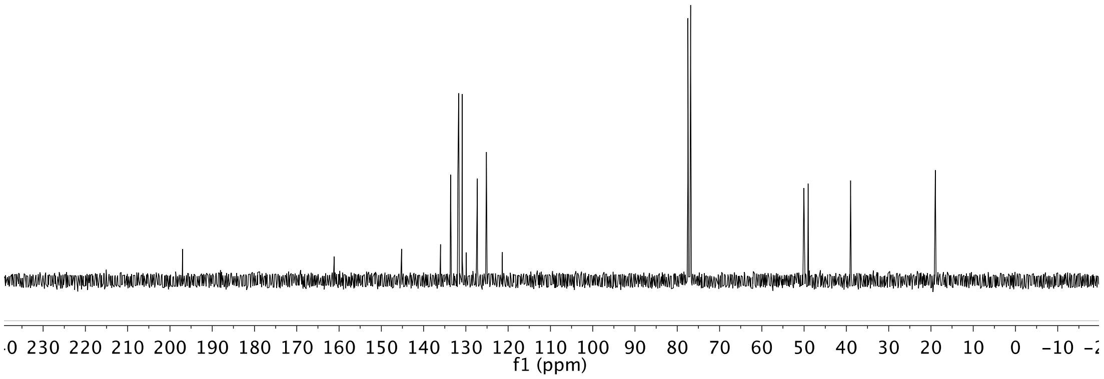

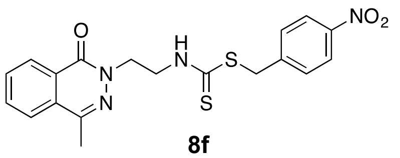
O
H
N
N
N
S
S
NO_2

8f

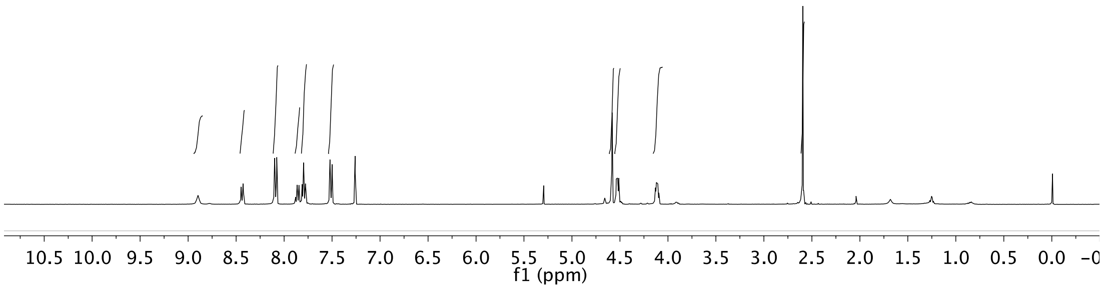
10.5 10.0 9.5 9.0 8.5 8.0 7.5 7.0 6.5 6.0 5.5 5.0 4.5 4.0 3.5 3.0 2.5 2.0 1.5 1.0 0.5 0.0
f1 (ppm)

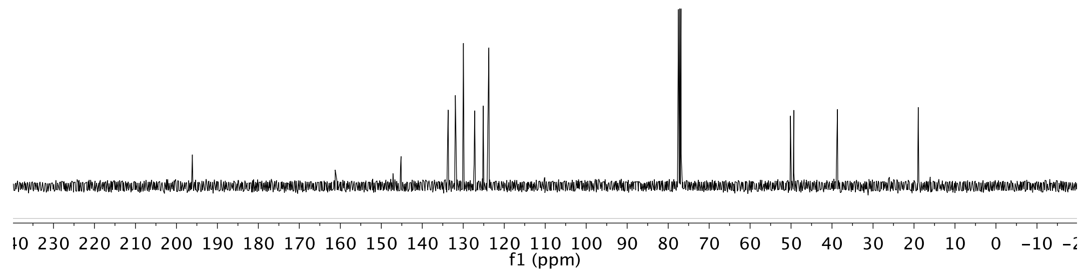
230 220 210 200 190 180 170 160 150 140 130 120 110 100 90 80 70 60 50 40 30 20 10 0 −10
f1 (ppm)

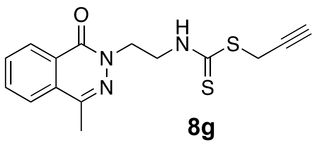
O
H
N
N
N
S
S

8g

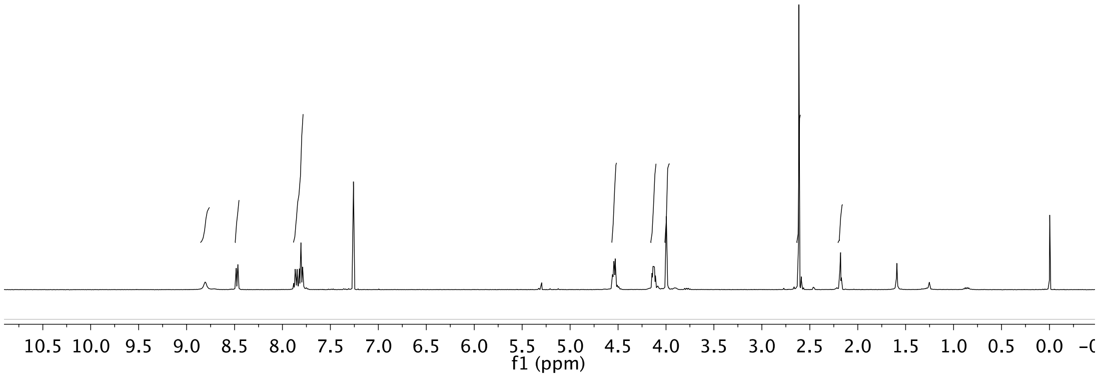
10.5 10.0 9.5 9.0 8.5 8.0 7.5 7.0 6.5 6.0 5.5 5.0 4.5 4.0 3.5 3.0 2.5 2.0 1.5 1.0 0.5 0.0
f1 (ppm)

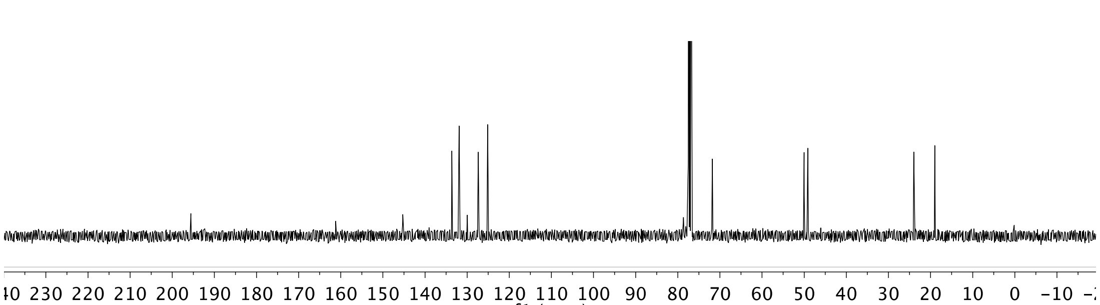
230 220 210 200 190 180 170 160 150 140 130 120 110 100 90 80 70 60 50 40 30 20 10 0 −10
f1 (ppm)

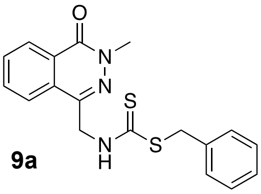

9a

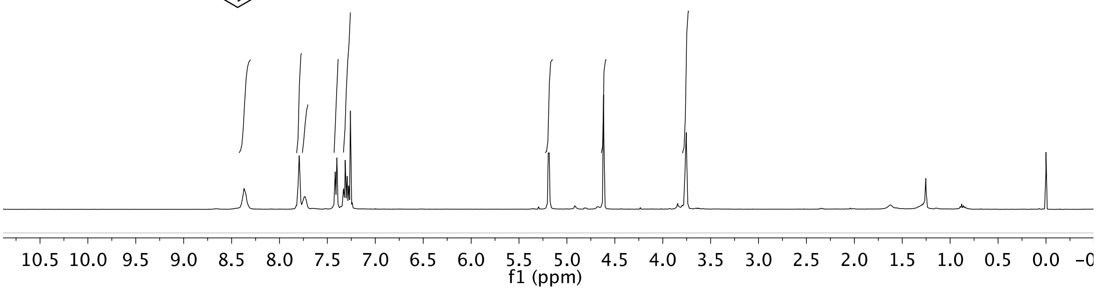
10.5 10.0 9.5 9.0 8.5 8.0 7.5 7.0 6.5 6.0 5.5 5.0 4.5 4.0 3.5 3.0 2.5 2.0 1.5 1.0 0.5 0.0
f1 (ppm)

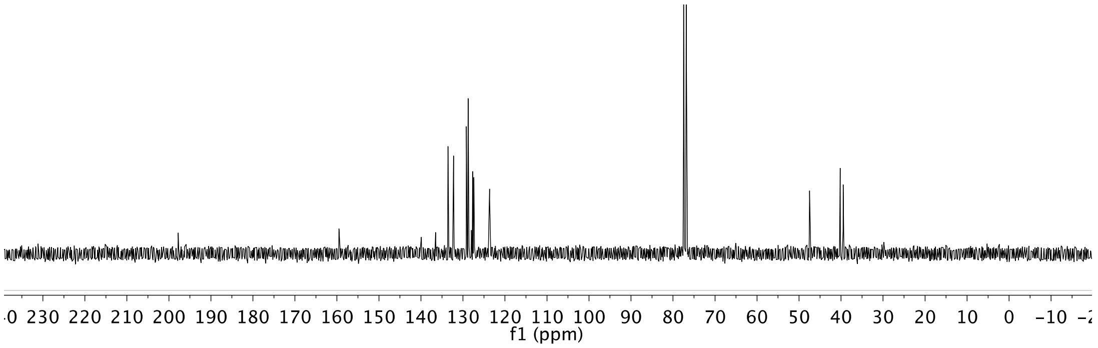
230 220 210 200 190 180 170 160 150 140 130 120 110 100 90 80 70 60 50 40 30 20 10 0 −10
f1 (ppm)

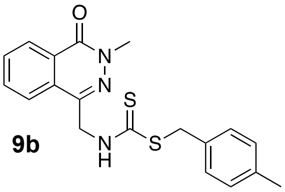

9b

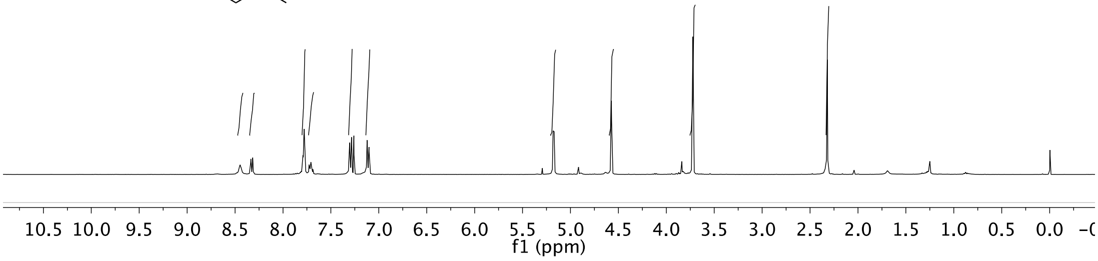
10.5 10.0 9.5 9.0 8.5 8.0 7.5 7.0 6.5 6.0 5.5 5.0 4.5 4.0 3.5 3.0 2.5 2.0 1.5 1.0 0.5 0.0
f1 (ppm)

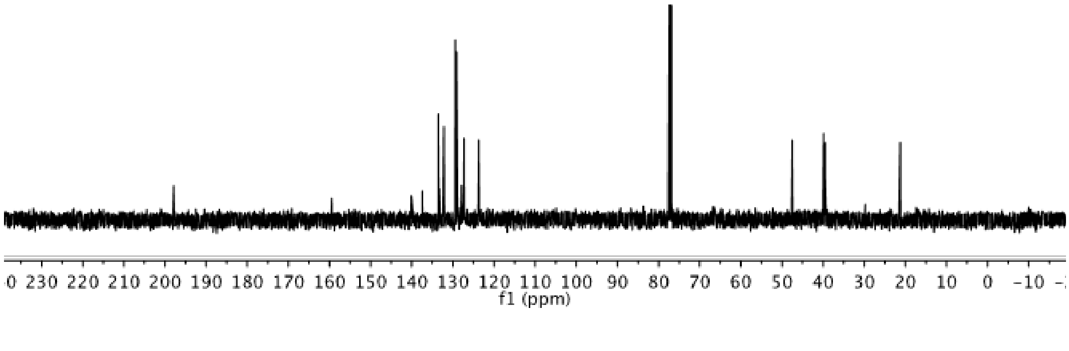
230 220 210 200 190 180 170 160 150 140 130 120 110 100 90 80 70 60 50 40 30 20 10 0 −10
f1 (ppm)

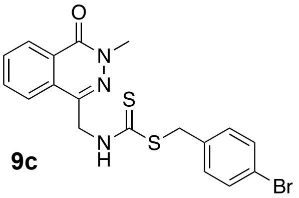
O
N
N
S
N
H
S
Br

9c

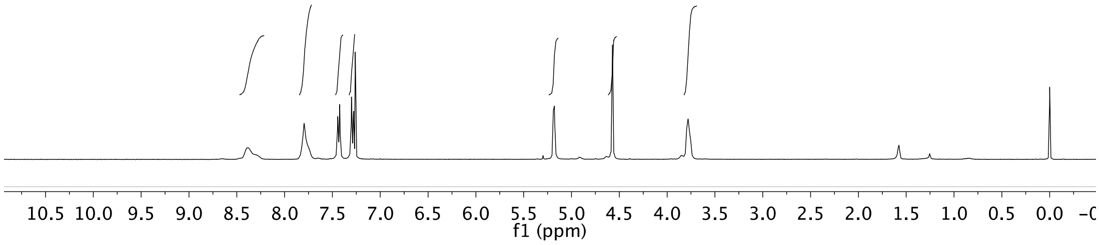
10.5 10.0 9.5 9.0 8.5 8.0 7.5 7.0 6.5 6.0 5.5 5.0 4.5 4.0 3.5 3.0 2.5 2.0 1.5 1.0 0.5 0.0
f1 (ppm)

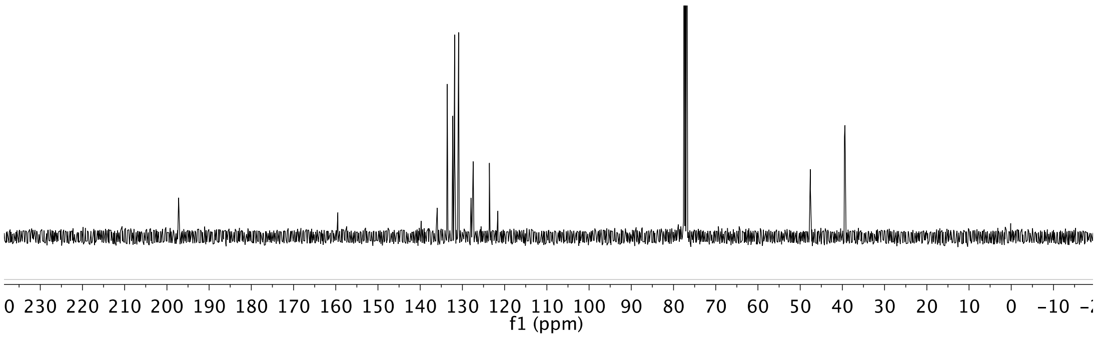
230 220 210 200 190 180 170 160 150 140 130 120 110 100 90 80 70 60 50 40 30 20 10 0 –10
f1 (ppm)

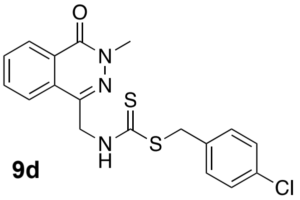
O
N
N
S
N
H
S
Cl

9d

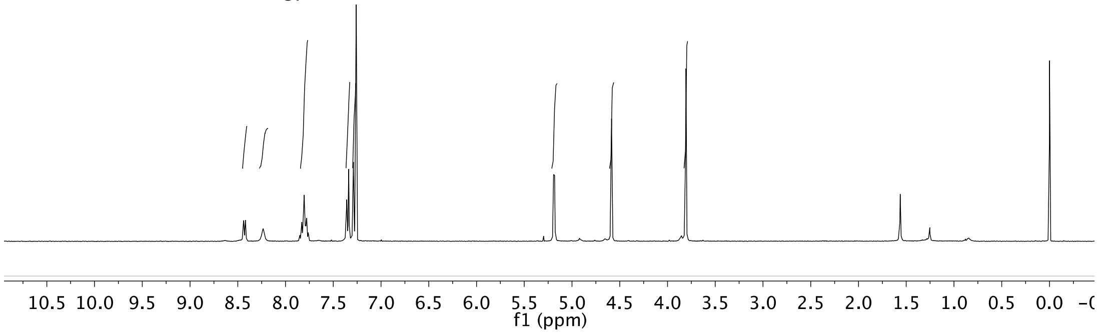
10.5 10.0 9.5 9.0 8.5 8.0 7.5 7.0 6.5 6.0 5.5 5.0 4.5 4.0 3.5 3.0 2.5 2.0 1.5 1.0 0.5 0.0
f1 (ppm)

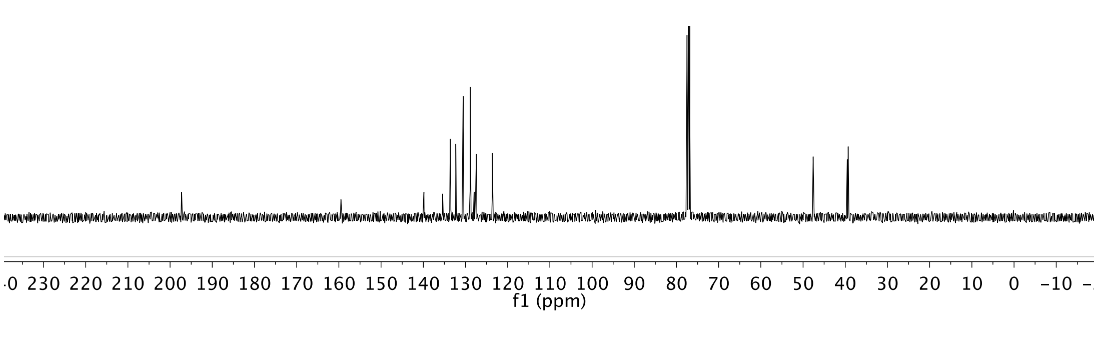
230 220 210 200 190 180 170 160 150 140 130 120 110 100 90 80 70 60 50 40 30 20 10 0 –10
f1 (ppm)

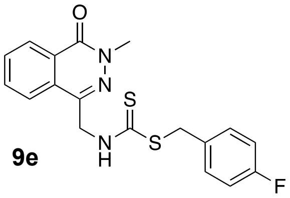

9e

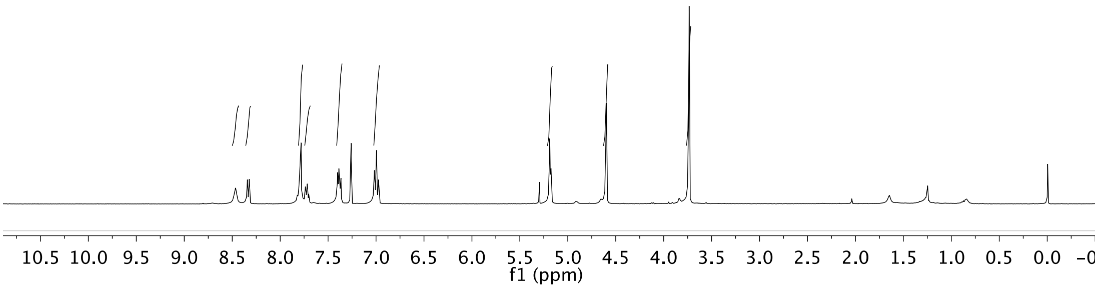

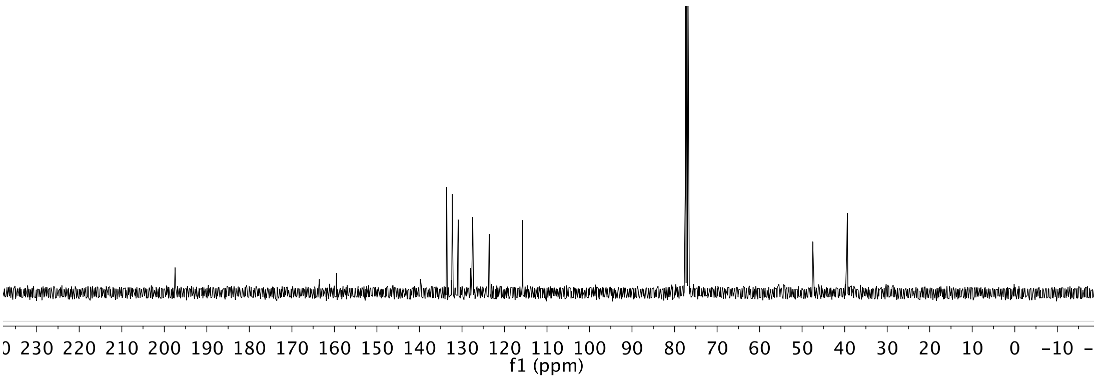

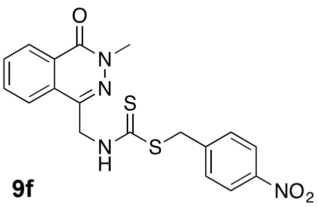

9f

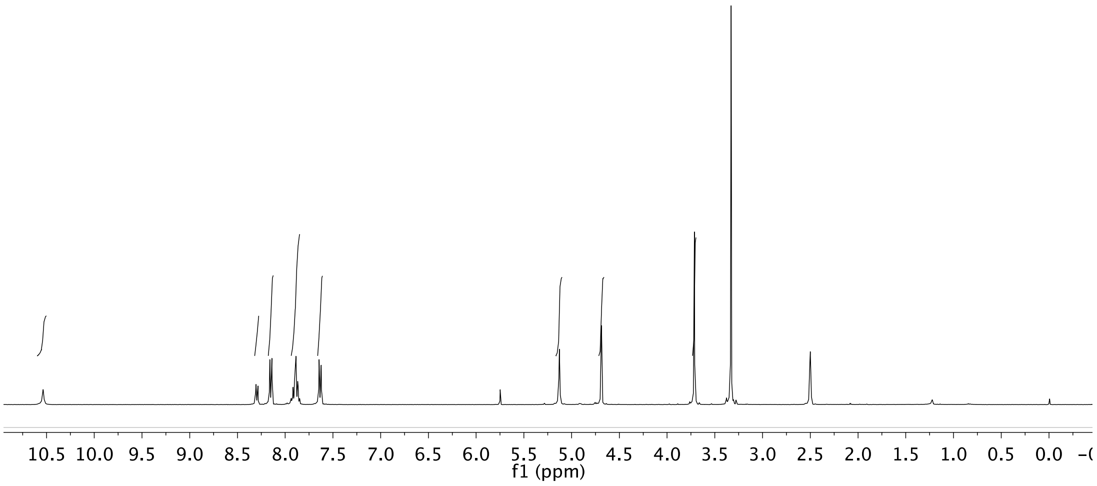

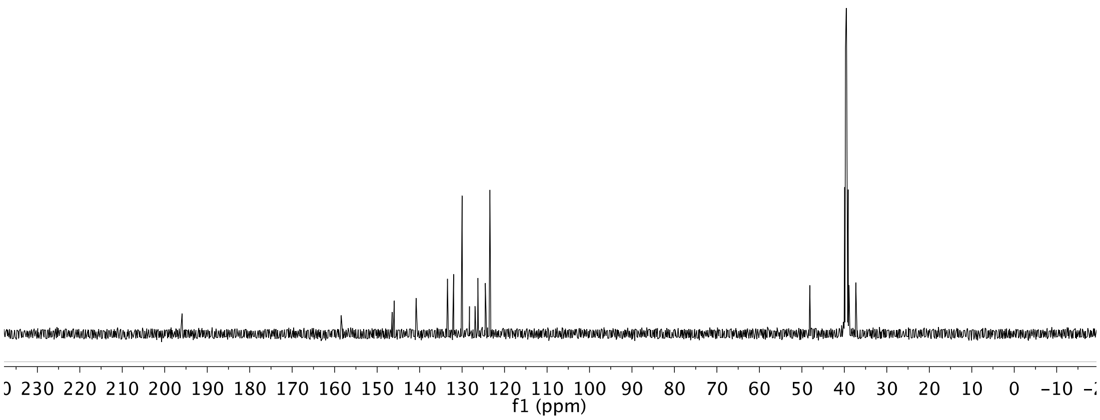
230 220 210 200 190 180 170 160 150 140 130 120 110 100 90 80 70 60 50 40 30 20 10 0 −10
f1 (ppm)

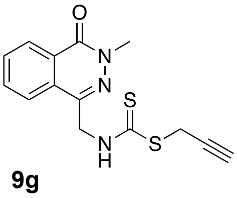
O
N
N
S
N
H
S

9g

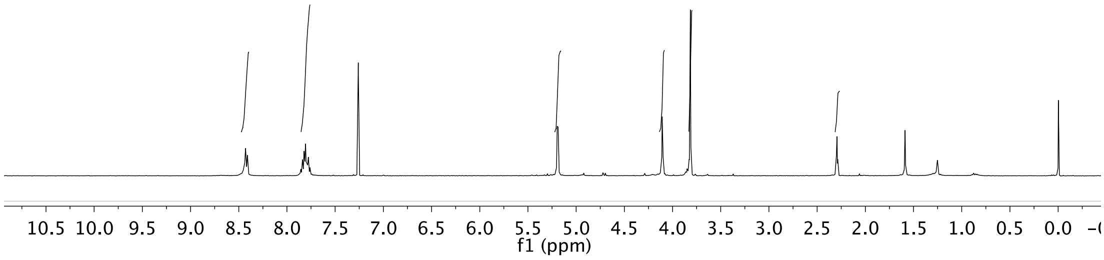
10.5 10.0 9.5 9.0 8.5 8.0 7.5 7.0 6.5 6.0 5.5 5.0 4.5 4.0 3.5 3.0 2.5 2.0 1.5 1.0 0.5 0.0
f1 (ppm)

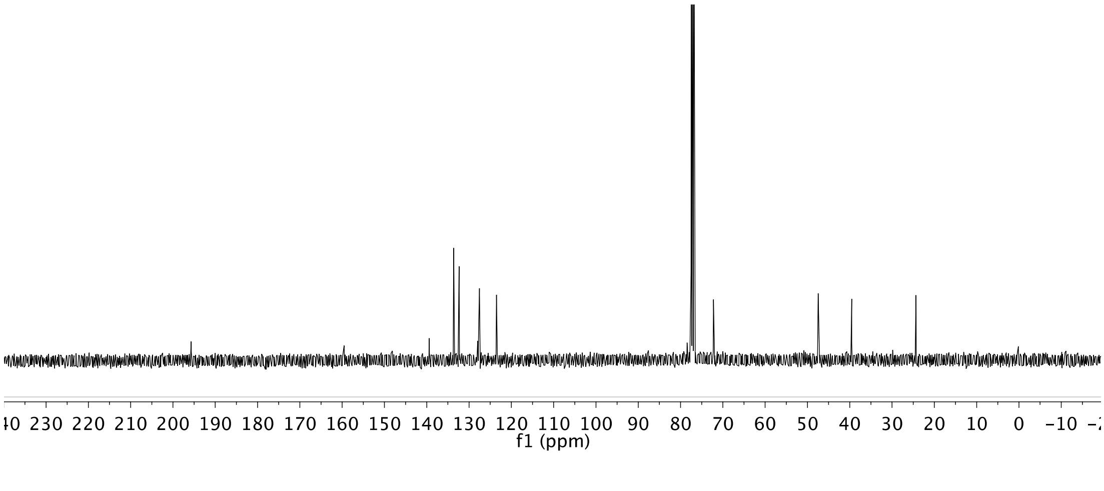
230 220 210 200 190 180 170 160 150 140 130 120 110 100 90 80 70 60 50 40 30 20 10 0 −10
f1 (ppm)
